# Relevance and consequence of economic and social resources of severely ill persons and their informal caregivers at the end-of-life: a systematic review of qualitative studies

**DOI:** 10.1186/s12904-025-01961-6

**Published:** 2025-12-12

**Authors:** Marlene Matzinger, Eleonore Baum, Daniela Bernhardsgrütter, Miriam Kesseli, Gerald Michelak, Fabiola Orosaj, Andrea Kobleder, Janine Vetsch

**Affiliations:** IGW Institute of Health Sciences, Eastern Switzerland University of Applied Sciences, Rosenbergstrasse 59, St.Gallen, 9001 Switzerland

**Keywords:** End of life, Economic resources, Social resources, Informal caregivers, Qualitative research

## Abstract

**Background:**

Life-limiting illnesses often lead to complex care needs and multifaceted support requirements for both severely ill persons and their informal caregivers, particularly at the end of life. The extent to which these needs are met may be significantly influenced by available economic and social resources. However, an in-depth understanding of how such resources shape end-of-life experiences is still missing. Therefore, our systematic review of qualitative studies aimed to explore and understand the perceptions of severely ill persons at the end of life and their informal caregivers regarding the relevance and consequences of their economic and social resources in varying settings of high-income countries.

**Methods:**

We followed the PRISMA guideline and searched MEDLINE via PubMed, CINAHL and PsychInfo from inception, which means the earliest available records in each database to October 2025. In addition, we searched reference lists of relevant studies. Three authors (DB, GM, MM) extracted the data, and four authors (DB, GM, FO, MM) assessed study quality using the JBI Critical Appraisal Checklist for Qualitative Research. The ratings were verified through mutual double checks. Any discrepancies were resolved through discussion with EB. We synthesized the data using qualitative content analysis according to Schreier.

**Results:**

In total, we included 20 studies reporting on various economic and social resources, which were sometimes interdependent. In the overarching category of economic resources, we identified three subcategories: (1) savings and assets, (2) workplace resources of informal caregivers, and (3) state support and insurance. The identified social resources could be structured into four subcategories according to House’s Social Support Theory: emotional support, appraisal support, instrumental support, and informational support.

**Conclusions:**

Severely ill persons and their informal caregivers face major emotional, practical, and organizational challenges at the end of life, and the extent of their burden is closely linked to available economic and social resources. Informal caregivers, who both provide and need support, require targeted professional and peer interventions to manage these demands and protect their well-being. Compassionate Communities appear to be a possible approach to reducing psychological burden, social isolation, and caregiver overload.

**Supplementary Information:**

The online version contains supplementary material available at 10.1186/s12904-025-01961-6.

## Background

Life-limiting illnesses pose difficulties for both severely ill persons and their informal caregivers, especially at the end of life (EOL). They may require complex care and have various support needs, along with a range of concerns. These support needs can include symptom management, emotional and spiritual support, assistance with daily and personal care, help with transportation, and communication with healthcare providers regarding disease progression and palliative treatment [1]. Economic and social resources may significantly influence the extent to which these needs are met [2, 3].

Economic resources - such as financial status, income and employment, health insurance, housing conditions, and material resources - can significantly affect the development of health or the onset of illness [4–6]. This is particularly relevant in the context of serious, chronic illnesses, where the burden of disease is often compounded by financial strain. At the EOL, severely ill persons and their informal caregivers face additional economic challenges, including loss of income or employment and high medical costs [7, 8]. Although in high-income countries existing health or social insurance systems often cover a large proportion of healthcare costs, severely ill persons and their informal caregivers still face high out-of-pocket expenditures. These additional expenses on healthcare tend to increase, particularly in the last year before death [9, 10].

In addition to the objective financial burden, the subjective perception of one’s financial situation is a key component of financial distress [11]. Financial distress can be significant and negatively impact health, even when, from a purely objective standpoint, sufficient economic resources are available to cover necessary expenses [12]. The systematic review by Walker-Pow et al. [10] shows that around a quarter of all participants living with terminal illness report experiencing high levels of financial insecurity at the EOL. Building on these findings, research in advanced cancer patients highlights the strong connection between financial difficulties and the experience of “total pain” [13, 14]. Coined by Cicely Saunders, the concept of “total pain” refers to the multidimensional nature of suffering at the EOL, encompassing physical, emotional, social and spiritual dimensions [15]. Financial hardship can acerbate suffering across all these four domains: it can worsen physical symptoms, increase emotional distress, strain social relationships, and negatively influence spiritual experiences. Consequently, financial distress is associated with worse outcomes across the full spectrum of total pain [13, 14]. Additionally, a recent systematic review shows that people with lower income are more likely to be admitted to and die in hospital than in home or hospice and may experience a lower quality of EOL care – even in high-income countries [16]. While supporting death at home is mentioned as a crucial part of palliative care policies, these policies overlook how this ideal aligns with populations with financial hardship [17, 18]. People experiencing financial hardship may have greater concerns about dying in poverty [19] and people living in socioeconomically disadvantaged areas tend to perceive greater benefit from EOL hospital admissions compared to those living in more affluent regions [20]. Furthermore, the possibility of spending the EOL at home depends not only on economic resources but also significantly on social resources [21].

Social resources are resources embedded within an individual’s social network and relationships, which can be accessed and used to achieve personal or collective goals [22]. A thematic synthesis of qualitative research reveals that social resources – specifically social support – is the main factor associated with access to the preferred place of death [23].

House [24] defines social support as a function of social networks, encompassing mutual assistance within these connections. His social support theory provides a structured understanding of how such connections translate into tangible forms of assistance to address individual needs and challenges. Studies suggest, that social support can impact how individuals assess stressful events and develop coping strategies, which in turn can reduce the physiological symptoms of chronic stress [25]. Palliative care interventions offering social support also show positive outcomes in both physical and psychological domains for patients with advanced cancer [26].

Despite growing recognition of the challenges faced by severely ill persons and their informal caregivers at the EOL, an integrated understanding of how economic and social resources shape EOL experiences is still missing.

The aim of this review is to explore and understand the perceptions of severely ill persons and their informal caregivers regarding the relevance and consequences of their economic and social resources at the EOL in varying settings of high-income countries. Gaining a deep understanding of these factors is essential for developing targeted support strategies that address the holistic needs of severely ill persons at the EOL and their informal caregivers.

## Methods

### Study design

We conducted a qualitative systematic review [27]. This type of review serves as an approach to synthesizing or comparing results from qualitative research. The qualitative systematic review seeks to identify “’themes’ or ‘constructs’ that lie in or across individual qualitative studies” [28]. The goal of a qualitative systematic review is interpretative, aiming to deepen the understanding of a particular phenomenon [27, 28]. We followed the PRISMA 2020 Checklist [29] to report this systematic review (Supplementary File 1).

### Eligibility criteria

We included articles if they met the criteria according to the PICo approach outlined by the Joanna Briggs Institute (JBI) [30] and reported the experiences of severely ill adult persons who were approaching the EOL. To allow for comparability we defined EOL similarly to the study conducted by Bowers et al. [31] as adults who are likely to die within the next 12 months according to the NICE guideline [32]. We defined persons to be severely ill, if they were diagnosed with an incurable advanced illness. The articles had to present the perspectives of the severely ill person approaching the EOL and/or the (bereaved) informal caregiver before and after the death of the ill person. The included articles had to be conducted in high-income countries as per the World Bank List of Economies 2025 [33] and needed to describe perceptions of economic and social resources relevant to the care situation at the EOL. Economic resources were defined as financial assets, income, employment and material conditions that influence persons’ access to essential services, caregiving and overall wellbeing [6]. Social resources were defined as any benefit, opportunity, or support derived from an individual’s position within a social network, including social ties, integration, and access to information, social capital, or assistance [34]. We included only empirical qualitative studies written in English, German, French, or Italian. No time restrictions were imposed. Table 1 shows the eligibility criteria according to PICo [30].

**Table 1:** Eligibility criteria according to PICo [30]

PICo components	Inclusion criteria	Exclusion criteria
Population: Severely ill persons and/or their (bereaved) informal caregivers at the EOL	Articles reporting the experiences of adult persons with a diagnosis of terminal illness who were approaching the EOL from the perspective of the ill person and/or from the perspective of the (bereaved) informal caregiver before or after the ill person passed away Studies including mixed populations were included only if the non-relevant population was a minority and results were reported separately for the population of interest	Articles reporting the experiences of severely ill persons or those under the age of 18 Articles reporting on the experiences of caregivers with children with a terminal illness Articles reporting the experiences of health professionals or social workers (as a proxy experience for severely ill persons or family members) Articles reporting the experiences of persons living with chronic illness and/or their family caregivers but not thought to be at the EOL Articles reporting the experiences of persons faced with “structural vulnerability” including (severe) poverty and homelessness (see definition from Stajduhar et al. [35])
Phenomenon of Interest	Articles which described perspectives and perceptions around economic and social resources that were relevant for the care situation at the EOL of severely ill persons	Articles that were focused on the place of death or on access to palliative care for people at the EOL including the availability of services in correlation to socioeconomic status and other measurement outcomes
Context	Research that was conducted in high income countries, as defined by World Bank List of Economies 2025 [33]	Low- and middle-income countries
Study design	Qualitative studies, mixed-methods studies (if qualitative results were reported separately)	Quantitative studies, reviews
Article type	Articles following the IMRaD structure (Introduction, Methods, Results and Discussion)	Grey literature, conference proceedings, unpublished work
Language	Articles in English, German, French and Italian	

### Information sources

We searched the following databases MEDLINE via PubMed, CINAHL and PsychInfo from inception, which means the earliest available records in each database until 21st of February 2024. We later re-ran the search on September 18th, 2024, to identify any newly published studies. In addition, we conducted a backward search by screening the reference lists of relevant studies to identify earlier studies. On October 15th, 2025, we conducted a further search update to include any additional studies published since the previous update.

### Search strategy

The search terms (Table 2) were developed, refined and agreed between EB, MK and AK, with support from JV. We used the components “end of life”, “economic resources”, “social resources” and “qualitative research” and identified relevant key words and medical subject headings, also from existing literature reviews [2, 23, 31]. We adapted the search string to each database’s specific syntax (Supplementary File 2).

**Table 2:** Search terms for PubMed

End of life [31]	AND	Economic resources [2, 31, 36]	AND	Social resources [2, 31]	AND	Qualitative research [23]
“Palliative Care“[Mesh]		“financial resource*”		“social resource*”		“Qualitative Research“[Mesh]
“Terminal Care“[Mesh]		“economic resource*”		“Social Capital“[Mesh]		Interview*
“Hospices“[Mesh]		“financial toxicity”		“Social Support“[Mesh]		**“**focus group*”
“Palliative Medicine“[Mesh]		“financial stress*”		“social capital”		Qualitative*
Terminal*		“financial strain”		“social support”		
Dying		“financial burden*”		“social class*”		
“Advanced illness*”		“financial hardship*”		Friend*		
“Life limit*”		“economic burden*”		Famil*		
EOL		income		relative*		
“Last year of life”		wage*		neighbor*		
EOLC		salar*		neighbour*		
“Advanced cancer”		savings		socioeconomic*		
“Advanced progressive illness”		“Financial Stress“[Mesh]		“socio-economic*”		
Hospice*		occupation				
Palliat*		job				
“end of life”		employ*				
“Hospice Care“[Mesh]						
“end-stage”						

### Selection process

First, we exported the search results from the individual databases into the citation management program Citavi software (version 7) [37], where duplicate records were identified and removed. After de-duplication, we uploaded the remaining results to the review management platform RAYYAN [38]. Four authors (EB, MK, DB, MM) conducted the screening of title and abstract independently and then retrieved the full texts for studies deemed eligible. We then went on to review them independently according to the eligibility criteria. In case of discrepancy or uncertainty, papers were read by AK, discussed in the team and a final decision made.

### Data collection process

We developed data extraction forms and included the following variables: author, year of publication, aim, setting, country, sample, type of illness, study design, study method and main findings. Three authors (DB, GM, MM) extracted data using Microsoft Excel. We verified the extracted data through mutual double checks.

### Critical appraisal

Four authors (DB, GM, FO, MM) rated the methodological quality of the included studies using the JBI Critical Appraisal Checklist for Qualitative Research [39]. We verified the ratings through mutual double checks. Any discrepancies were resolved through discussion with EB. This JBI-Checklist consists of 10 items which evaluate the quality and methodological rigour of the data by answering with “yes”, “no”, “unclear” and “not applicable”. We did not exclude any articles from this literature review due to poor methodological quality.

### Data analysis

MM and DB analyzed the data using qualitative content analysis according to Schreier [40] supported by MAXQDA software (version 2024) [41]. Following Schreier’s approach, we applied a combination of deductive and inductive coding. In a first step, MM thoroughly reviewed the study results and highlighted key sections. Subsequently, MM and DB deductively developed overarching categories ‘economic resources’ and ‘social resources’. We further applied House’s framework of social support [24] to deductively categorize social support into four distinct types, emerging from both natural (e.g. family, friends) and formal support systems (e.g. health professionals) [42]:

- Emotional support, including comfort, companionship, appreciation, and encouragement,
- Appraisal support, which involves feedback on behavior,
- Informational support, such as providing guidance on addressing challenges or identifying sources of help, and.
- Instrumental support, such as practical assistance (e.g., helping with shopping for bedridden individuals, providing transportation, or childcare).

During the initial coding, one author (MM) coded the study results section by section according to these deductively developed main categories. The initial coding was double checked by DB. Subsequently, both authors (MM, DB) developed subcategories inductively based on the data, allowing new patterns to emerge that were not anticipated in the deductive framework. All identified subcategories were discussed and consolidated into broader categories, with definitions provided for each. All study results were then re-coded by MM and DB using the developed coding frame.

## Results

**Fig. 1:**
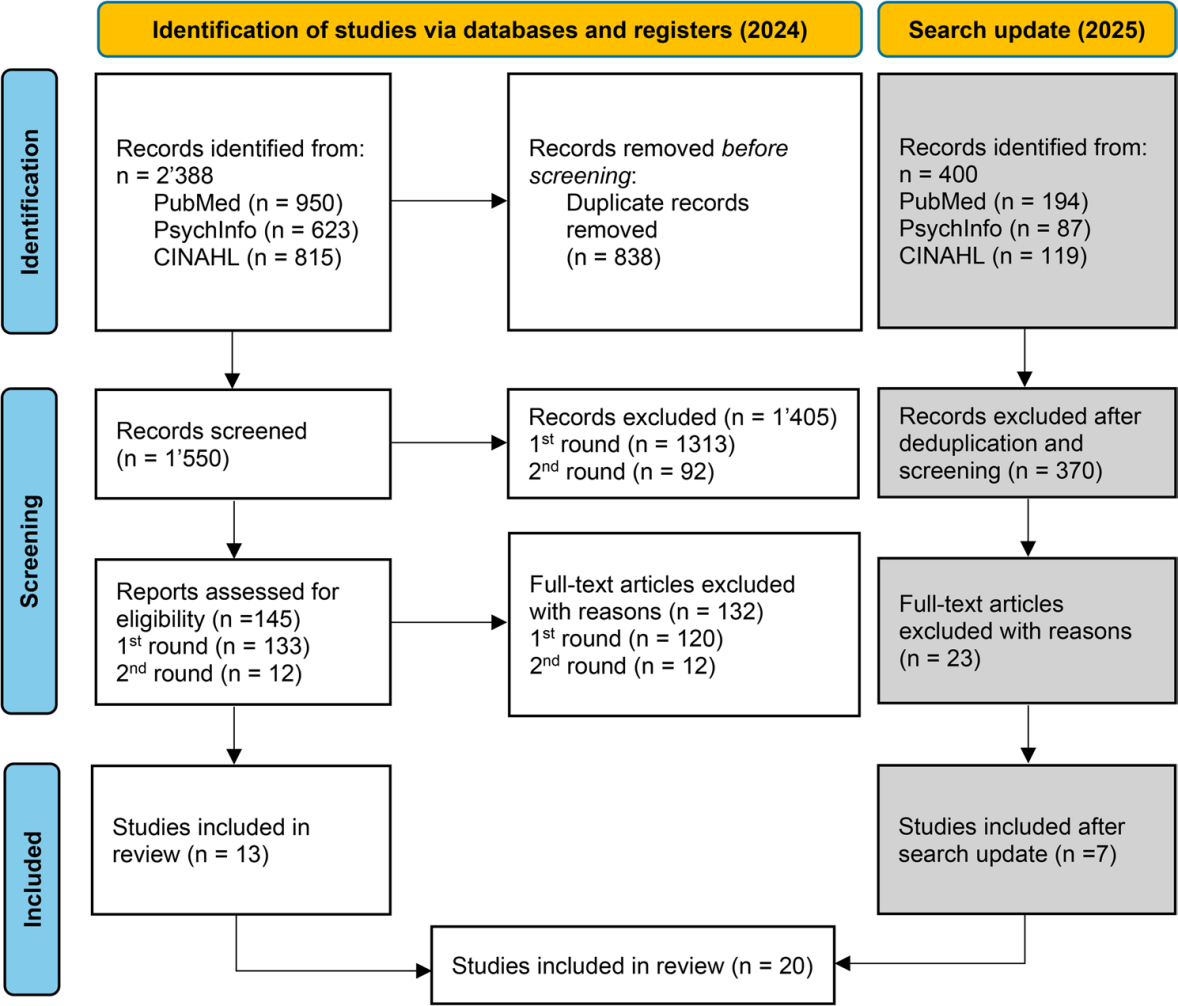
PRISMA flow diagram (adapted, including search update)

In total, we identified 2,388 studies in the initial search with an additional 400 new records from the search from September 2024 to October 2025. After the removal of duplicates, we screened 1,550 studies in 2024 and 305 in the search update 2025 (see Fig. 1). Initial screening of titles and abstracts resulted in 145 eligible papers for full-text review in 2024 and 30 records in 2025. A total of 13 studies from the initial search and 7 studies from the search update were included in the review. Overall, this review includes 20 studies (see Table 3).

**Table 3:** Summary of included studies

Author, year of publication	Aim	Setting, country	Sample, type of illness	Study design, study method	Main findings
Ang et al. (2025) [43]	To comprehend and interpret the lifeworld experiences of Chinese Singaporean family caregivers who provide EOL cancer care for their loved ones at home.	Home hospice setting in Singapore	10 Chinese Singaporean family caregivers (age 37–81) of adult cancer patients at EOL	Hermeneutic phenomenology In-depth interviews with field notes of objective observations	Economic resources: Workplace resources for informal caregivers: adjusting work schedule to find time for caregiving responsibilities. Social resources: Emotional support: feelings of guilt for not doing enough or being present; maintaining positivity while caring; reassurance from healthcare professionals. Appraisal support: caregiving transformed life perspectives and supported coping; knowing the patient’s last wishes provided preparedness; spirituality or faith facilitated coping; talking about EOL topics depending on cultural aspects. Instrumental support: helping the severely ill with routine activities; frustration and helplessness when unable to help; sharing caregiving responsibilities to reduce stress and burden; caregiving led to a transformation in informal caregivers’ life perspective that supported coping effectively with their responsibilities.
Bijnsdorp et al. (2021) [46]	To provide in-depth insight into the experiences and support needs of working informal caregivers of patients with a life-threatening illness, barriers and facilitators in the combination of work and family care, and how this affects informal caregivers.	Home-based EOL care Netherlands	18 working informal carers (age 30-70 years) of patients with life-threatening illness (dementia, cancer, stroke, organ failure, progressive neurodegenerative disorder)	Longitudinal qualitative study Individual interviews Online questionnaire for demographic data	Economic resources: Workplace resources for informal caregivers: job changes, taking care leave, having autonomy/flexibility at work, reducing work hours, working remotely, having supportive/understanding colleagues/supervisors, adapting work tasks, early retirement improved the caregiving situation; not knowing about care leave, combining work and care increased burden. Social resources: Emotional support: talking with friends/family/other informal caregivers, going to work to relieve tension; scheduling free time was difficult. Instrumental support: arranging respite care/upscaling professional healthcare provision to improve the care situation; seeking help from psychologists; support from volunteers; sharing care tasks with family members was positive but also caused tension; caregiving gave a sense of fulfillment but also puts pressure on the informal caregiver; staff shortage and less flexibility increased pressure on informal caregivers. Informational support: bad communication and lack of guidance led to higher burden as well as bureaucracy/unclear application procedures.
Bijnsdorp et al. (2022) [45]	To describe the trajectories in the burden for working informal caregivers of patients with a life-threatening illness, and to determine which factors in paid work and care are related to changes in burden over time.	Home-based EOL care Netherlands	17 working informal carers (age 30-70 years) of patients with life-threatening illness (dementia, cancer, stroke, organ failure, progressive neurodegenerative disorder)	Longitudinal qualitative study Individual interviews Online questionnaire for demographic data	Economic resources: Workplace resources for informal caregivers: job changes, stable income, supportive supervisor/colleagues, having autonomy/flexibility at work, working fewer hours/no nightshifts/weekend shifts, working remotely, adapting work tasks, knowing about/taking care leave decreased burden; self-employment, pressure at work, negative experiences with supervisors/colleagues increased burden; informal caregivers returning to work quickly to show gratitude. Social resources: Emotional support: changing healthcare team over time led to burden; sharing situation with fellow sufferers, psychological help reduced burden; informal carers supported care recipients by general presence. Instrumental support: moving to a care facility decreased burden but informal caregivers felt guilty also when accepting outside help; shared caregiving and having an easy to access network decreased burden. Informational support: receiving information from libraries or the Internet, open/clear communication with healthcare providers decreased burden; lack of aftercare/guidance from healthcare team and bureaucracy increased burden.
Close et al. (2021) [47]	To explore the experiences of managing everyday legal needs among people approaching the EOL and their informal caregivers.	Hospice and primary care North-East England	14 informal caregivers (mean age 67.8 years) and 13 patients (mean age 70.3 years) with cancer, COPD, dementia, neurodegenerative or neuromuscular diseases	Qualitative study Semi-structured interviews Patient/ caregiver dyads interview	Economic resources: Workplace resources for informal caregivers: previous working status was a barrier to ask for financial help. State support and insurance: feeling ashamed to rely on external resources. Savings and assets: worries that costs could escalate/run out of control; gaining financial resources supported informal caregivers’ quality of life. Social resources: Instrumental support: healthcare professionals helping finalizing plans and providing information about social welfare legals generated relief; difficulties for informal caregivers to get information and support. Informational support: friends/family members with medical background providing support. Appraisal support: talking about the future and planning helped care recipients, but some struggled with talking about the EOL.
Coristine et al. (2003) [54]	To describe the psychosocial impact on caregivers of caring for women with advanced breast cancer and to learn about caregivers’ experiences.	Home-based EOL care Canada	12 spouse caregivers (SCGs, age 35-85 years) and 6 non-spouse caregivers (NSCGs, age 35-55 years) No data provided on the illness	Qualitative study Focus groups with bereaved caregivers	Economic resources: Workplace resources for informal caregivers: facing time conflicts with combining work and care; getting self-employed to fit employment hours around caregiving hours, flexible supervisors were helpful. State support and insurance: covering medications through extended health insurance plans or participation in clinical trials. Savings and assets: covering medication by self-payment; limited financial resources made it difficult to get medication when needed. Social resources: Emotional support: cooperation between family members; informal caregivers as advocates for care recipients. Instrumental support: moving the care recipient to an institution/new environment decreased burden of informal caregivers but wasn’t wished by them; care recipients wished to move to a care facility; getting support from general care services and household help meant giving up; sharing caregiving tasks helped but also led to tension between family members; informal caregivers accompanying and transporting patients to appointments and providing caregiving tasks. Informational support: healthcare professionals providing information, open/clear communication with service providers. Appraisal support: healthcare team providing decision-making support.
Dillon et al. (2024) [60]	This study’s goal was to provide in-depth understanding of how the organization and fragmentation of healthcare impacts the experiences of patients with advanced cancer and their families, especially near the EOL.	Not-for-profit healthcare organization in Northern California, the Palo Alto Medical Foundation (PAMF) USA	281 patients (mean age 68 years), 24 family members Patients with advanced cancer, stage IV disease at diagnosis or distant metastases	Mixed-methods quality improvement study Set of open-ended patient survey questions, in-depth interviews	Economic resources: Savings and assets: not paying for medication/treatments because it is too expensive. State support and insurance: financial burden despite having insurance; rejected claims and paying out-of-pocket led to “shock and surprise”; complex insurance rules; frequent insurance denials despite eventually reversed led to stress. Social resources: Emotional support: importance of advocates, such as family members. Informational support: need for help to coordinate care between clinical teams, specialties, institution, delivery systems; lack of communication between teams and institutions; discontinuity of care led to dissatisfaction of care; having access to better information on costs; not knowing/getting informed about high costs in advance; not understanding rules for insurance coverage led to incurred expenses which could have been avoided; additional costs and frustration due to scheduling timely primary care; palliative care teams coordinating care; patients and families experiencing administrative burden (spending significant time and energy on organizing medical care, prescriptions, insurance approvals).
Dunsmore-Dawson (2020) [48]	To explore the experiences of caregivers for patients who were accessing palliative care services for an illness diagnosed as terminal or life limiting.	Home-based EOL care UK	9 caregivers (age 40-78 years) providing EOL care No data provided on the illness	Phenomenological approach Longitudinal qualitative study Individual interviews	Economic resources: Workplace resources for informal caregivers: having good pension, taking care leave, care recipients employing the informal caregiver. State support and insurance: financial support through state support. Savings and assets: using savings to pay formal caregivers. Social resources: Emotional support: having trust in and making friends with healthcare professionals; talking to too many healthcare professionals confused informal caregivers; sharing caregiving experiences to feel encouraged; informal caregivers as advocates for care recipients; importance of social networks; shrinking social world due to deteriorating health status of the care recipient. Instrumental support: hospice as a safe shelter, cooperating with different clinical services/service providers positively impacted psychological health; support network for informal caregivers and care recipients; shared family caregiving; feeling exhausted because of all the things to do. Informational support: open/clear communication with paid carers and service providers; having family members/friends with background in healthcare; bureaucracy as burden for informal caregivers. Appraisal support: feeling powerless/frustrated because of not being included in the decision-making process.
Enyert et al. (1999) [57]	To assess the caregivers’ sense of emotional well-being and their ability to transcend and find meaning in the caregiving experience.	Home-based EOL care USA	7 informal caregivers (age 49-68 years) whose family members had died within the last six months to one year of the study. No data provided on the illness	Qualitative study Ethnographic techniques and grounded theory methodology Individual interviews	Economic resources: Workplace resources for informal caregivers: supportive supervisor. State support and insurance: receiving state support, paying bills through health insurance; care recipient not believing in life insurance led to informal caregiver experiencing bankruptcy. Social resources: Emotional support: sharing caregiving experiences in bereavement groups, support from social networks (family members/friends/church community) was valued by the informal caregivers. Instrumental support: splitting caregiving with siblings. Informational support: healthcare professionals provided support.
Essue et al. (2015) [9]	To prospectively measure out-of-pocket costs of medical and health-related care and to explore the factors that contribute to economic hardship from patients’ and caregivers’ perspectives.	Specialist palliative care program in a community setting Australia	30 patients (mean age 68 years) and 22 informal caregivers No data provided on the illness	Mixed-method study – prospective case study Individual interviews 2-week-diary	Economic resources: Workplace resources for informal caregivers: working remotely, having an understanding boss; unawareness of leave option stressed informal caregivers/ care recipients, ceased/reduced employment income led to financial hardship. State support and insurance: private health insurance to pay care, government-funded income support was too less to pay rent/utilities. Savings and assets: using savings/borrowing money to face economic hardship/ pay for care; ability to remortgage the house; care recipients not owning a house struggled with paying rent/utilities; strategies to save money: extensions with bill collectors cutting down services; transportation costs consumed income support. Social resources: Informational support: too complex forms to fill out for palliative care support and allowances/governmental funded income support.
Giesbrecht et al. (2015) [55]	To identify socio-environmental factors that contribute to palliative informal caregiver resilience in the Canadian homecare context.	Home-care nursing in the palliative and chronic care context Canada	16 families of which 11 care recipients were palliative and 6 chronic No data provided on the age of participants and illness	Qualitative case study; secondary data analysis Semi-structured individual interviews with family caregivers, patients	Economic resources: Savings and assets: no need to pay mortgage, freedom to install required equipment or make structural renovations; flexibility by owning a vehicle. Workplace resources for informal caregivers: working remotely or part-time, being seasonally employed, being retired. Social resources: Instrumental support: having access to different clinical services and service providers, shared family caregiving, supportive family members.
Hanratty et al. (2012) [49]	To explore the views of older adults who are receiving health and social care at the end of their lives, on how services should be funded, and describe their health-related expenditure.	Secondary care setting specialized on lung cancer, heart failure or stroke North-West-England	30 older adult patients (age 69-93 years) with lung cancer, heart failure or stroke	Qualitative study Individual interviews	Economic resources: Savings and assets: people with financial resources available should contribute to or pay for care; assets/savings/home should not be sacrificed to pay for care; informal caregivers bridging gaps between complex and slowly responsive funded care and expense of self-funding. Social resources: Instrumental support: shared family caregiving. Informational support: through healthcare professionals.
Herbst et al. (2023) [50]	To gain an understanding of the specific EOL support experiences and needs of patients and caregivers.	Home-based EOL care of informal caregivers Germany	Terminally ill adult children (age 27-73 years), parent carers (age 64-85 years), terminally ill parents (age 36-89 years), adult child carers (age 23-66 years); 65 patients with (non-) cancer, 42 carers	Mixed-methods study Grounded theory approach Embedded quantitative self-report questionnaires in semi-structured qualitative interviews	Economic resources: Workplace resources for informal caregivers: work commitments were practical restrictions for informal caregivers, care recipients supporting the informal caregiver financially. Social resources: Emotional support: need for psychosocial support/pastoral care for patients to cope with the illness; informal caregivers wishing for exchange between care recipients, informal caregivers supporting care recipients by general presence and attention. Instrumental support: hiring household help; accepting outside help was difficult for informal caregivers (feeling guilty over personal limitations); informal caregivers providing care and administrative support. Appraisal support: patients protecting family members by withholding or concealing information about their illness.
Isenberg et al. (2021) [56]	To explore and understand patients’ and caregivers’ expectations and experiences of the hospital-to-home transition while receiving palliative care.	Hospital-to-home transition and palliative care Canada	14 informal caregivers (mean age 60 years) and 25 patients (mean age 66 years) with cancer, stroke, dementia or pulmonary fibrosis	Longitudinal, prospective, qualitative study Grounded theory approach Individual interviews and patient-caregiver dyads	Economic resources: Savings and assets: paying additional private care because government-funded care was not sufficient; medical costs, auxiliary costs like transportation led to limited financial resources Social resources: Emotional support: “caring” palliative care physicians, church community supporting informal caregivers and patients, family/friends support improved wellbeing of informal caregiver and patient. Instrumental support: patients feeling safe in hospitals due to continuous care; support through professional and paid services. Informational support: healthcare professionals educating practical skills and about symptoms; being overwhelmed because of poor communication with hospitals and service providers.
Kreyer et al. (2024) [52]	To identify which support needs were discussed with the family caregivers and the types of support delivered to meet these needs.	Seven specialized palliative home care teams Austria	484 informal caregivers No data provided on the age of the participants and the illness	Implementation study Retrospective analysis of electronic records with quantitative and qualitative methods	Economic resources: State support and insurance:need for financial support for caring aids, bathroom remodeling. Workplace resources of informal caregivers:need for the possibility to reduce working hours and care leaves. Social resources: Emotional support:psychological support; informal caregivers seeking contact to a self-help group; support to handle family conflicts and communication problems within the family; support for informal caregivers to deal with inner personal conflicts and burdensome situations. Informational support:Advice and information about the illness, managing symptoms, what to expect in the future (e.g. expected symptoms, preventive measures, support services), accessing equipment and support/respite services, legal matters, work issues, financial support. Instrumental support:practical training to enable informal caregivers to manage symptoms and to provide personal care (e.g. incontinence/wound care) for the severely ill person; feelings of burden due to diminishing personal resources, being solely responsible for caregiving and due to other responsibilities (e.g. balancing their own family); practical household help; health care professionals coordinating care services. Appraisal support: help from health care professionals to talk about death and dying; round tables; reassuring; support regarding illness perception and decision-making; support for spiritual concerns.
Lewis et al. (2011) [61]	To explore and describe the socioeconomic demographics, needs, capacities and experience of EOL care for patients and/or carers from this most disadvantaged area evidenced by: - The social, economic and care outcomes at individual, community and government levels. **- **The nature and impact of social capital outcomes. **- **The experience of access to a specialist palliative care service.	Community-based specialist palliative care service in a socio-economically disadvantaged area Western Sydney, Australia,	16 severely ill persons (mean age 66.3 years) with advanced, life-limiting diseases (mostly malignant) and 6 informal caregivers (mean age 56.8 years) from a lower socio-economic background. 2 severely ill persons declined to be interviewed	Concurrent embedded mixed methods design with qualitative emphasis In-depth semi-structured interviews	Economic resources:Savings and assets: limited savings made large expenses difficult; income loss forced asset sales and cheaper housing; reduced spending due to financial uncertainty; funeral plans prepared for future costs.State support and insurance: relying on state-funded funerals/early superannuation release/welfare benefits; burdensome out-of-pocket costs despite Medicare/subsidies; trusting yet inflexible welfare systems.Workplace resources of informal caregivers:Long-term caregiving limited job options, causing income loss/stress; working part-time/from home to manage future financial uncertainty. Social resources: Emotional support: close family/friend relationships supported self-esteem, small or strained family networks increased stress; social networks provided emotional support; good communication and patient-centered care fostered trust and respect for palliative care staff.Informational support: financial/care-related advice from professionals/family members with medical expertise; palliative care settings provided clear, accessible information; welfare agencies were helpful but required demanding applications. Instrumental support: Specialist palliative care nurses provided crisis support and service coordination, but limited referrals restricted access; helpful community nursing, though, offered minimal hands-on care. Appraisal support: Church groups as important source to meet spiritual needs vs. irrelevance of religion; recognition/reassurance from formal caregivers/agencies strengthened confidence in managing care demands.
Lim et al. (2024) [44]	To explore the occupations of community-dwelling terminally ill Chinese older adults and their caregivers living in Singapore	Home and daycare services of a community-based palliative care provider	16 terminally ill older persons (mean age: 76 years) with cancer and non-cancer diagnosis and their informal caregivers (mean age 63)	Qualitative exploratory approach Individual and joint interviews	Economic resources Savings and assets: informal caregivers reported financial constraints but supported the severely ill person’s financial needs; lack of affordable portable oxygen tanks as a barrier to participation. Workplace resources of informal caregivers:worry about leaving severely ill persons alone at home while balancing work and caregiving. Social resources: Emotional support: informal caregivers supported emotional needs but experienced stress; limited participation of severely ill persons (e.g. dependence in toileting); informal caregivers as gatekeepers of participation; participation to avoid disappointing family (e.g., dining out); caregivers sought leisure/social activities while prioritizing care; informal caregiving motivated by a commitment to the relationship, by reciprocity, and avoiding regret; informal caregivers encouraged their families to express their love and concern while the severely ill person was still alive. Instrumental support: depending on family members or domestic helpers (e.g., transport) to access the community; informal caregivers provided all physical and practical care, supervised domestic helpers, and faced role strain; some severely ill persons accepted help, others still stived for independence. Appraisal support: reframing and accepting uncontrollable situations; mindset of acceptance helped value each day; death preparation and communication varied.
Pieper et al. (2025) [51]	To investigate the financial impact of caring for a seriously ill and dying family member in Germany by using a panel.	Home-based EOL care Germany	304 family caregivers (mean age 50.5 years) of deceased persons with cancer, neurological disease, cardiovascular disease and/or respiratory disease	Online observational panel survey with open-text options	Economic resources: Workplace resources of informal caregivers:employer flexibility/flexible working hours;taking sick leave/leave of absence for care because of lack of options for time off; having understanding employers; in need for financial compensation due to loss of work while caring for a seriously ill relative. State support and insurance: in need for comprehensive cost coverage by the care insurance funds; in need of financial and social protection to provide care without compromising the informal caregiver’s health; insufficient resources make arranging professional care difficult; the classification into care levels often did not reflect the relative’s actual needs, leading to frustration and dissatisfaction. Social resources: Informational support: lack of knowledge and guidance about available support, both financial and care related; in need of better support in navigating bureaucracy due to frustrating interactions with authorities; in need of assistance with paperwork and applications.
Sacchi et al. (2025) [53]	To explore the factors influencing the decision-making process regarding the place of dying and death involving migrant cancer patients in Italy.	Palliative Care unit of the Azienda USL-IRCCS of Reggio Emilia Italy	28 migrant patients with advanced metastatic cancer (lung, pancreatic, biliary tract, and thyroid cancer), family members, cultural mediators, funeral operators, social workers, and key informants	Grounded theory Semi-structured interviews	Economic resources: Savings and assets: lack of essential medications and high costs of treatments in home countries/treatments are inaccessible without significant financial resources; high costs for travelling in the home country; concerns about high costs of posthumous repatriation creating financial strain on families. State support and insurance: patients did not receive state disability-related financial support due to lack of a valid residence permit; not everyone can afford insurance. Social resources: Emotional support: community solidarity provided relief in financially hard situation; presence of family members. Appraisal support: lack of clarity or open discussion about the terminal stage of illness as well as delayed or absent cultural/linguistic mediation and bureaucratic complexities impeded the decision-making process; family members’ protective stance leads to lack of transparency about terminality; faith traditions provide existential reassurance and continuity. Informational support: lack of clear, centralized pathways for obtaining essential information; not knowing where to seek guidance forced to navigate multiple offices and institutions; cultural mediators, charitable organizations and funeral service providers played a crucial role in alleviating bureaucratic burdens, helping families navigate complex systems.
Solomon et al. (2013) [58]	To explore the experiences of one patient who died at home and to interpret how dying at home influenced patterns of bereavement for patient’s family.	Home-based EOL care, where the participant made the decision to die at home USA	a 78-year-old female diagnosed with amyotrophic lateral sclerosis six months prior to death, her husband and her 3 children	Qualitative study; single case study Individual interviews	Economic resources: Savings and assets: ability to give gifts to those leaving behind (material gifts); being able to afford formal caregivers relieved burden. Social resources: Emotional support: having confidence in the healthcare team; family members supporting each other’s in ways they didn’t know before, open communication between family members leads to comfort, intimacy and safe environment at home helped care recipient feeling loved/safe, dying at home offered ideal environment for anticipatory loss and bereavement. Instrumental support: shared family caregiving. Informational support: having necessary information when needed. Appraisal support: healthcare professionals supporting autonomy in decision-making process.
Waldrop et al. (2005) [59]	To understand how caregivers make the transition to end-stage caregiving and to illuminate its unique aspects using a stress process model.	Home-based EOL care USA	74 informal caregivers (age 21-87 years) No data provided on the illness	Qualitative study Individual interviews	Economic resources: Workplace resources: employed informal caregivers struggling with combining work and care because of inflexible work schedules, unsupportive co-workers increased burden, informal caregiver left jobs because of fulltime caregiving. Social resources: Emotional support: finding strength in renewed and deepened family relationships, support from church community. Instrumental support: organizing general care services as support, informal caregivers providing administrative support for the care recipient. Informational support: getting information through internet/libraries or healthcare professionals/family members with medical background. Appraisal support: informal caregivers getting support through belief and religion; rejection of religious involvement, anger at God for allowing the illness.

All included studies were conducted in high-income countries, namely Singapore (*n* = 2) [43, 44], the Netherlands (*n* = 2) [45, 46], United Kingdom (UK) (*n* = 3) [47–49], Germany (*n* = 2) [50, 51], Austria (*n* = 1) [52], Italy (*n* = 1) [53], Canada (*n* = 3) [54–56], the United States of America (USA) (*n* = 4) [57–60] and Australia (*n* = 2) [9, 61].

14 out of 20 studies were qualitative studies [43–49, 53–59]. The qualitative designs were varied: two studies with a phenomenological approach [43, 48], two with a grounded theory approach [53, 56], one study used ethnographic techniques and grounded theory methodology [57], two studies were case studies [55, 58] and one study used a qualitative exploratory approach [44]. Six studies did not describe the design specifically [45–47, 49, 54, 59]. One study was mixed-methods [9], another study was mixed-methods with a grounded theory approach [50] and one study was a mixed-methods quality improvement study [60]. One study had a concurrent embedded mixed methods design with a qualitative emphasis [61], one study was an implementation study [52] and one study conducted an online observational panel survey with open-text option [51].

Nine studies conducted individual interviews [9, 45, 46, 48, 49, 56–59] and one study individual and joint interviews [44]. Five studies stated the conduction of (in-depth) semi-structured interviews [47, 50, 53, 55, 61] and one study conducted focus group interviews [54]. One study conducted in-depth interviews with field notes of objective observations [43] and one study in-depth interviews and a set of open-ended patient survey questions [60]. Demographic data was collected through questionnaires in two studies [45, 46]. In one study participants filled out a two-week-diary with services used [9] and one study collected data by observing homecare nurses during visits at severely ill persons’ homes [55]. One study used embedded quantitative self-report questionnaires [50]. Six studies [45, 46, 48, 54, 57, 59] interviewed only informal caregivers and one study [49] only severely ill persons. One study conducted a retrospective analysis of electronic records with qualitative and quantitative methods [52] and one study used open-text options in an online observational panel survey [51].

Nine studies included participants with various diagnoses [44–47, 49–51, 56, 61], three studies patients with (advanced) cancer [43, 53, 60], one study included only one participant with amyotrophic lateral sclerosis [58] and seven studies did not report the underlying diseases of the participants [9, 48, 52, 54, 55, 57, 59].

Most of the studies (*n* = 13) focused on persons receiving home-based palliative care and EOL care [43–46, 48, 50–52, 54, 55, 57–59]. Three studies focused on inpatient palliative care and EOL care [49, 53, 60] and three on palliative care and EOL care in community and primary care settings [9, 47, 61]. One study examined the transition from hospital to home [56].

### Critical appraisal

Table 4 shows the quality appraisal synthesis of the included studies according to the JBI Critical Appraisal Checklist for Qualitative Research [39]. In all studies, the methodological approaches were transparently reported, allowing for a clear understanding and replication of the results but only seven studies [43, 44, 48, 52, 53, 60, 61] provided information on the researchers’ cultural and theoretical stance, while also reflecting on the reciprocal influence between the investigators and the study outcomes. The detailed and complete quality appraisals for each individual study can be found in Supplementary File 3.

**Table 4:** Quality appraisal synthesis

Study	Item 1 Congruity between the stated philosophical perspective and the research methodology	Item 2 Congruity between the research methodology and the research question or objectives	Item 3 Congruity between the research methodology and the methods used to collect data	Item 4 Congruity between the research methodology and the re-presentation and analysis of data	Item 5 Congruence between the research methodology and the interpretation of results	Item 6 Locating the researcher culturally or theoretically	Item 7 Influence of the researcher on the research, and vice-versa, is addressed	Item 8 Representation of participants and their voices	Item 9 Ethical approval by an appropriate body	Item 10 Relationship of conclusions to analysis, or interpretation of the data	Inclusion/Exclusion
Ang et al. 2025 [43]	Yes	Yes	Yes	Yes	Yes	Yes	Yes	Yes	Yes	Yes	Inclusion
Bijnsdorp et al. 2021 [46]	Yes	Yes	Yes	Yes	Yes	No	No	Yes	Yes	Yes	Inclusion
Bijnsdorp et al. 2022 [45]	Yes	Yes	Yes	Yes	Yes	No	No	Yes	Yes	Yes	Inclusion
Close et al. 2021 [47]	Yes	Yes	Yes	Yes	Yes	No	No	Yes	Yes	Yes	Inclusion
Coristine et al. 2003 [54]	Yes	Yes	Yes	Yes	Yes	No	No	Yes	Yes	Yes	Inclusion
Dillon et al. 2024 [60]	Yes	Yes	Yes	Yes	Yes	Yes	Yes	Yes	Yes	Yes	Inclusion
Dunsmore-Dawson 2020	Yes	Yes	Yes	Yes	Yes	Yes	Yes	Yes	Yes	Yes	Inclusion
Enyert et al. 1999 [57]	Yes	Yes	Yes	Yes	Yes	Yes	Unclear	Yes	Yes	Yes	Inclusion
Essue et al. 2015	Yes	Yes	Yes	Yes	Yes	No	No	Yes	Yes	Yes	Inclusion
Giesbrecht et al. 2015	Yes	Yes	Yes	Yes	Yes	Unclear	No	Yes	Yes	Yes	Inclusion
Hanratty et al. 2012 [49]	Yes	Yes	Yes	Yes	Yes	No	No	Yes	Yes	Yes	Inclusion
Herbst et al. 2023 [50]	Yes	Yes	Yes	Yes	Yes	Unclear	No	Yes	Yes	Yes	Inclusion
Isenberg et al. 2021 [56]	Yes	Yes	Yes	Yes	Yes	No	No	Yes	Yes	Yes	Inclusion
Kreyer et al. 2024 [52]	Unclear	Yes	Yes	Yes	Yes	Yes	Yes	Yes	Yes	Yes	Inclusion
Lewis et al. 2011	Yes	Yes	Yes	Yes	Yes	Yes	Yes	Yes	Yes	Yes	Inclusion
Lim et al. 2024 [44]	Yes	Yes	Yes	Yes	Yes	Yes	Yes	Yes	Yes	Yes	Inclusion
Pieper et al. 2025 [51]	Unclear	Yes	Yes	Yes	Yes	No	No	Yes	Yes	Yes	Inclusion
Sacchi et al. 2025 [53]	Yes	Yes	Yes	Yes	Yes	Yes	Yes	Yes	Yes	Yes	Inclusion
Solomon et al. 2013 [58]	Yes	Yes	Yes	Yes	Yes	No	No	Yes	Yes	Yes	Inclusion
Waldrop et al. 2005 [59]	Yes	Yes	Yes	Yes	Yes	No	No	Yes	No	Yes	Inclusion

### Relevance and consequences of economic and social resources

**Fig. 2:**
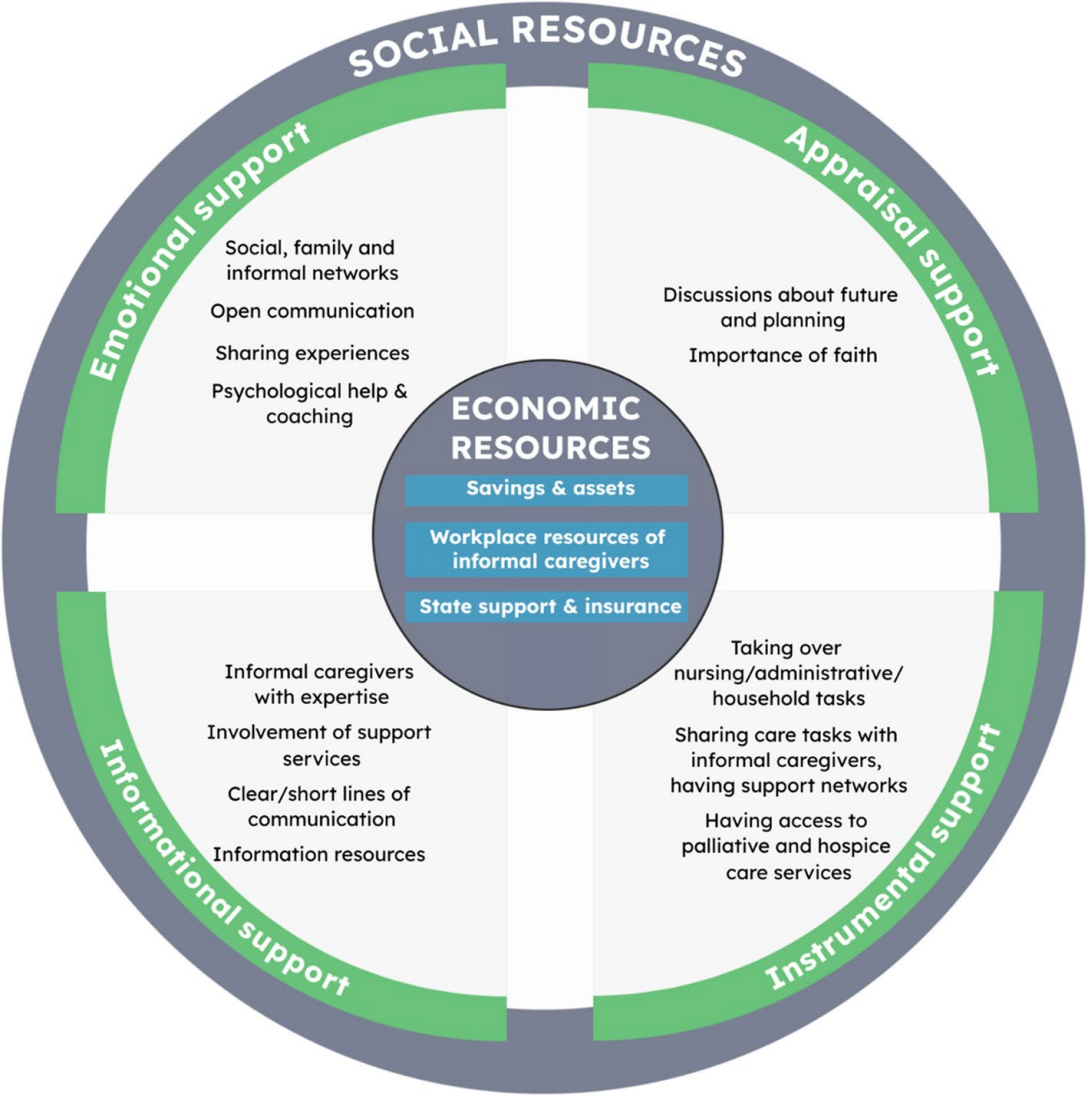
Structure of the review results

Figure 2 presents the structure of our review results based on the overarching categories of *economic resources* and *social resources*. The category of economic resources encompasses both financial and material resources available to severely ill persons and their informal caregivers (*savings and assets*), as well as external economic support provided through government assistance and insurance schemes (s*tate support and insurance*). Additionally, *workplace resources* that offered support to informal caregivers like adjustments in employment, opportunities to participate in shaping the workplace situation and supportive colleagues and supervisors are part of the economic resources. Within the category of *social resources*, we classified the identified forms of support into four subcategories – *emotional support*, *appraisal support*, *informational support*, and *instrumental support* – based on House’s social support framework [24]. Emotional support encompasses social networks, family members and informal caregivers as well as open communication, sharing experiences and psychological help and coaching programs. Appraisal support includes discussions about the future and planning as well as the importance of faith. In the category of informational support, important resources included informal caregivers, family members or friends with expertise as well as the involvement of support services, clear and short lines of communication and other information resources like libraries or the Internet. Instrumental support comprises informal caregivers taking over nursing, administrative, and household tasks, sharing care tasks with other family members and having support networks as well as access to palliative and hospice care services.

### Economic resources

#### Savings and assets

Savings and assets are considered fundamental components of the economic resources available to severely ill persons and their informal caregivers [9, 44, 47–49, 53–56, 58, 60, 61]. Savings were used to pay formal carers and medication and to balance the loss of income that some caregivers had and reduce burden [9, 48, 54, 58, 61]. Some severely ill persons and informal caregivers intentionally contributed to funeral plans as a way to prepare for future financial obligations [61]. In one study [49] both informal caregivers with and without financial resources held beliefs, that individuals with financial resources available should contribute to or pay for their health and social care. In contrast, some of the participants also mentioned that they did not want to sacrifice their savings, assets and homes to pay for care [49]. In one study [58] the severely ill person described that the ability to give material gifts to those being left behind was an important way to create a dying environment that was ideal.

Having adequate financial resources to ensure care positively impacted the informal caregivers’ psychological health. It also made a significant difference to their perceived quality of life [47]. Economic resources also had an impact on participation in daily activities (e.g. lack of affordable oxygen tanks as a barrier) [44].

In the main theme of “savings and assets” studies observed that financial hardship led severely ill individuals and informal caregivers to miss payments and medical appointments, to fail filling prescriptions [9] and avoid paying for medication or treatments because they were too expensive [60]. Both, severely ill persons and their informal caregivers developed strategies like cutting down services, letting insurance and private health care policies expire, delaying medical appointments and negotiating extensions with bill collectors to avoid service disruption to save money [9, 61]. Furthermore, borrowing money helped facing financial hardship [9]. Financial hardship was exacerbated when essential medications and treatments were unavailable or unaffordable in migrant care recipients’ home countries, forcing them to spend significant financial resources to access care. High travel costs within the home country and concerns about the financial burden of posthumous repatriation further strained the severely ill person and their informal caregivers [53]. 

Another important economic resource for severely ill persons and their informal caregivers during the caregiving period was having assets like cars or houses [55]. Some severely ill persons and/or informal caregivers who owned houses had the freedom to buy and install required equipment and make structural renovations if that deemed necessary [55, 56]. Also the possibility of taking out a mortgage on the house or not having to pay off a mortgage was seen as a resource by the severely ill persons and their informal caregivers [9, 55]. Owning vehicles helped them to be flexible [55].

Severely ill persons who lived in rented accommodations sometimes struggled with paying for the rent and could not afford utilities or much needed medication on time [9, 54]. The raising medical costs, private care and auxiliary costs as well as additional costs such as expenses for transportation also led severely ill persons to fear that costs could escalate and run out of control [9, 47, 56].

#### Workplace resources of informal caregivers

Workplace resources played an essential role in enabling informal caregivers to carry out their EOL care duties [9, 43–48, 50–52, 54, 55, 57, 59, 61]. The combination of work and caregiving tasks was perceived as burdensome by many informal caregivers, leading to changes in employment: Some had to reduce their working hours to provide the necessary care which often led to financial hardship [9, 43, 52, 61]. Several informal caregivers were also in need of financial compensation due to the loss of work while caring for a seriously ill relative [51]. For some informal caregivers, being retired allowed them to be flexible without losing income or facing financial consequences [48, 55]. However, early retirement was not an option for most informal caregivers due to financial reasons [46]. In some cases, informal caregivers left their job because of the full-time caregiving duties [59, 61], searched for another job because they were unable to reconcile the current work and care demands [45, 46] or became self-employed which, in turn, increased their burden [45]. Informal caregivers experienced limitations in job choices because of long term care giving demands [61]. Some severely ill persons wanted to support the informal caregiver financially and employed the family member as an informal caregiver [48, 50].

In contrast, several studies also revealed that, despite the challenges, some caregivers were able to combine both roles successfully [45–47, 50, 54, 59]. What helped informal caregivers to reconcile work and care was being involved in shaping the workplace situation [9, 45, 46, 48, 51, 54, 55, 57, 59]. Having flexible working tasks and hours [45, 46, 51, 59], being able to work remotely [9, 45, 46, 55], having autonomy at work [45, 46, 54], working part-time and being seasonally employed were also perceived as positive resources [55]. Several studies mentioned the possibility to take unpaid or paid care leave [9, 45, 46, 48]. However, the unpredictable illness trajectory made it difficult to decide when to take the leave [45, 46]. Other informal caregivers did not know about the possibility to take care leave or mentioned that there were insufficient care leave schemes at their workplace [9, 45, 46, 52]. Some also required sick leave/leave of absence for care because there were no adequate option for taking time off [51]. Supportive colleagues and a supportive boss helped decreasing the burden in informal caregivers [9, 45, 46, 54, 57, 59]. Feeling listened to and understood by the supervisor and arrangements with colleagues about trading or taking over shifts improved the work-care-situation [9, 45, 46, 51, 54, 57]. One study reported that the employer gave informal caregivers time off for a longer period without loss of salary or formal arrangements to care for the severely ill person at the EOL [45]. After the death of their loved ones, informal caregivers returned quickly to work to show gratitude for the support [45, 48] and because it gave them energy and satisfaction [45]. In contrast, unsupportive supervisors and co-workers who could not understand the demands of caregiving increased burden and led to burnout symptoms in some informal caregivers [45, 54, 59].

#### State support and insurance

State support and insurance were highlighted as crucial economic resources accessible to severely ill persons and their informal caregivers [9, 47, 48, 51–54, 57, 60, 61]. In some cases, informal caregivers applied and got assessed for funding to cover their caregiving costs [48]. They received support from the government or through a council to cover the costs for changing needs in severely ill persons and secure caring at home [48, 54, 57]. Access to welfare fostered trust in government institutions, but the inflexibility of the benefit system posed a challenge [61]. Several informal caregivers reported the need for comprehensive cost coverage by care insurance funds, noting that existing schemes often fell short of actual care needs [51]. In addition, some patients did not receive state disability-related financial support because they did not have a valid residence permit, and not everyone could afford insurance [53].

Despite having access to subsidies for living and medical expenses, informal caregivers struggled to pay rent and necessary utilities because the government-funded income support was inadequate and insufficient, and they experienced economic hardship [57]. In addition, they faced a substantial financial burden despite having insurance: rejected claims and out-of-pocket payments often led to “shock and surprise,” while complex insurance rules and frequent insurance denials, even when eventually reversed, caused additional stress [60]. Limited financial resources also made it difficult to arrange professional care, further increasing the strain on informal caregivers [51].

Informal caregivers and severely ill persons expressed the need for financial support for caring aids or for building modifications such as bathroom remodeling [52]. They emphasized the need for stronger financial and social protection to provide care without compromising their own health [51]. Some severely ill persons and informal caregivers felt ashamed to rely on external financial resources or mistakenly believed that their previous employment would make them ineligible for support, which discouraged them from seeking help [47]. The classification into care levels often did not reflect the relative’s actual needs, leading to frustration and dissatisfaction among informal caregivers [51]. Some severely ill persons and informal caregivers used additional financial and insurance support, such as government funded health insurance programs, private or extended health insurance [9, 54, 57] or the participation in clinical trials to cover medication costs [54].

### Social resources

#### Emotional support

Our findings highlight the importance of social networks for severely ill persons and their informal caregivers. Throughout the caregiving period, talking to others outside the caregiving scenario and connecting with family, friends and established relationships provided informal caregivers with a sense of normality and helped them to find meaning beyond their caregiving role [46, 48, 57, 61]. Informal caregivers acted as gatekeepers of participation [44] and developed coping strategies in identifying resources in local (support) groups or communities, like church communities [48, 50, 56, 57, 59, 61]. All these informal support networks were considered as valuable systems for helping to cope with the caregiving situation [48, 56, 61]. Additionally, community solidarity provided relief in financially hard situations [53]. 

Although social networks were important for severely ill persons and their informal caregivers their social world was becoming increasingly restricted [44, 48, 61]. Reasons were the severely ill persons’ reduced state of health or the lack of free time for informal caregivers due to their caregiving responsibilities [44, 48, 61]. 

Family members and informal caregivers were a vital resource to provide emotional support to the severely ill person as advocates or by their general presence [43, 45, 48, 50, 53, 54, 60, 61]. They helped the care recipient to deal with anxiety and depression [54]. Married partners and informal caregivers who shared the same illness trajectory and treatment path as the care recipient were especially important sources of emotional support [54]. The intimacy between the severely ill person and the informal caregiver along with the privacy and familiar environment at home, was described as a situation that made the severely ill person feel comfortable, safe and improved well-being [56, 58]. It also offered informal caregivers the opportunity to process their own anticipatory loss and bereavement [58]. They provided care out of commitment to the relationship, gratitude toward the care recipient, a desire to reciprocate care, and a wish to avoid living with any regrets [44].

Spending too little time with the care recipient sometimes left informal caregivers feeling guilty [43, 45]. However, they wished for (more) opportunities for the care recipients to exchange with other terminally ill persons because they felt emotionally exhausted from providing support [50]. Therefore, it was important for informal caregivers to help themselves emotionally by setting clear boundaries [45] and receiving emotional support from family members, who were not looking after the care recipient. Informal caregivers found strength in renewed and deepened family relationships [59]. 

Open communication [44, 46, 58, 61] and new forms of cooperation between family members [54, 58] provided emotional support to informal caregivers. Sitting together with loved ones and talking about feelings was a valuable resource for them [59] and helped through the grieving process [57]. However, family conflicts and communication problems within the family posed an additional burden on severely ill persons and informal caregivers and underscored the need for professional support [52].

Another big emotional support resource for informal caregivers were other informal caregivers to share their experiences. Talking about the situation with others in similar circumstances was helpful: it eased their feelings of isolation and made them realize how exhausting caregiving could be [45, 48, 52]. Even though, sometimes informal caregivers were in need of more professional support. 

Psychological help and coaching programs helped to decrease the burden in informal caregivers and severely ill persons coping with the illness [45, 50, 52]. Having trust and confidence in the healthcare team also supported informal caregivers and care recipients emotionally [47, 58, 61]. Sometimes they developed friendships with the members and staff of healthcare institutions [48, 61]. The consistency of the caregiving team and the presence of a primary contact person were crucial and provided a sense of relief [45, 47, 48].

#### Appraisal support

The importance of appraisal support was evident in discussions about the future and planning. Knowing the severely ill person’s last wishes helped informal caregivers feel prepared [43]. Professionals played a key role as enablers and initiators of discussions about illness perception and about the future [47, 48, 52]. Decision-making support from various healthcare professionals was important for both severely ill persons and informal caregivers [47, 52]. Not being involved in future planning could be frustrating and burdensome for informal caregivers [48]. A lack of clarity or open discussion about the terminal stage of illness, as well as delayed or absent cultural/linguistic mediation and bureaucratic complexities, often impeded the decision-making process [53]. In addition to professional support, conversations with family and friends provided crucial opportunities for severely ill persons to reflect on one’s wishes regarding the future [47]. Previous experience in supporting family, friends, or neighbors with life-limiting illnesses empowered some severely ill persons to successfully complete their future planning [47].

However, conversations about the EOL were not always easy neither for severely ill persons nor their family caregivers and also depended on cultural aspects; some withheld information about their illness and prognosis to protect the other party [43, 47, 50, 52, 53]. Spirituality or faith as a kind of appraisal support could facilitate informal caregivers’ coping with the impending EOL [43]. Faith traditions provided existential reassurance and a sense of continuity for some participants [53]. However, the importance of faith was viewed controversially [52, 59, 61]. For some informal caregivers and severely ill persons, belief in God appeared to be helpful in coping with the impending EOL. Others rejected religious involvement or even expressed anger at God for allowing the illness to happen [59]. 

#### Informational support

The high level of bureaucracy (e.g. insurance approvals, organizing medical care) placed a significant burden on informal caregivers [9, 45–48]. In some cases, care recipients themselves also experienced this form of administrative strain [60] and reported needing better support in navigating bureaucracy due to frustrating interactions with authorities, as well as assistance with paperwork and applications [51]. Cultural mediators, charitable organizations, and funeral service providers played a crucial role in alleviating bureaucratic burdens, helping families navigate complex systems [53]. Having support through healthcare professionals and support providers and clear and short lines of communication with them were very important for informal caregivers and severely ill persons [46, 48, 49, 52, 54, 57, 59, 61]. They provided information about the severely ill persons’ healthcare status and demanding medical support, the disease and progression and played a critical role in identifying work-related and social welfare issues [47, 50, 52, 59, 61]. They also explained therapeutic effects and side effects of medication and provided guidance on the next steps when curative treatments stopped [46, 54]. The awareness of available support services helped prevent informal caregivers from experiencing burnout [45, 55] and access to healthcare professionals increased their sense of control [54, 59]. Severely ill persons and informal caregivers valued the clear communication within palliative care and perceived it as a supportive environment for asking questions [61].

Furthermore, informal caregivers played a vital role in keeping the surrounding informed about what was going on [45]. Especially informal caregivers, family members or friends with expertise about the disease or general medical knowledge as well as knowledge of the healthcare system provided informational support for the severely ill persons in understanding their disease and healthcare path, putting things in perspective and navigating through the possibilities of support [45, 47, 48, 55, 58, 59]. For the care recipient, they functioned as “ears” and “translators”, as one study mentioned [54]. But not only knowledge about the disease was important: informal caregivers also supported the severely ill persons by gaining information about legal needs [47] and involving support services if necessary.

Despite this, informal caregivers faced challenges due to lack of communication and coordination between clinical teams, institutions, and service providers, which sometimes led to discontinuity of care and dissatisfaction with the services provided. Informal caregivers also reported difficulties in obtaining information on healthcare costs, not being informed in advance about high costs, and understanding rules for insurance coverage. A lack of understanding in these areas often resulted in avoidable expenses [60]. Informal caregivers further reported the lack of clear, centralized pathways for obtaining essential information. Not knowing where to seek guidance often forced them to navigate multiple offices and institutions [53]. Additionally, informal caregivers reported a lack of knowledge and guidance about available support, both financial and care-related [51]. The need for informational support and guidance extended beyond the death of the care recipients. However, informal caregivers reported that this kind of aftercare from health professionals was lacking [45, 46].

Furthermore, poor or absent communication, along with the lack of a suitable time or place to speak to healthcare professionals, negatively impacted the relationships between informal caregivers, severely ill persons and healthcare professionals. This increased the burden and feelings of being overwhelmed [45, 46, 48, 56]. Furthermore, unsatisfying communication with municipalities or other organizations was burdensome [45, 46]. Therefore, some of the informal caregivers drew on a range of other information resources like libraries or the internet [48, 59].

#### Instrumental support

Informal caregivers provided many different forms of instrumental support for their care recipients [43–50, 52, 54–59, 61]. They supported them by assisting with or taking over nursing tasks such as giving injections or changing dressings and were in charge for medication [50, 52, 54]. Some informal caregivers supervised domestic helpers [44]. They also helped the care recipient with administrative duties such as paying bills, selecting insurance, writing wills and organizing the severely ill persons’ burial [50, 59]. Furthermore, informal caregivers took over more household tasks such as cooking or cleaning [54]. In some cases, they even redesigned the home to make it more accessible for the severely ill person [48]. Others played a vital role in transporting and accompanying severely ill persons to their medical appointments and social activities [44, 54]. In some cases, caregiving transformed participants’ outlook on life, fostering a positive perspective rooted in their beliefs and ideologies, which in turn helped them cope more effectively with their responsibilities [43]. However, when providing these forms of instrumental support informal caregivers sometimes experienced stress [45] or role strain [44], especially when taking over nursing tasks [54]. Informal caregivers frequently felt exhausted from managing multiple tasks simultaneously and keeping them in mind [48, 52] and reported feelings of frustration and helplessness when they were unable to provide support [43].

One instrumental support resource for informal caregivers was sharing care tasks with family members [43, 45, 46, 49, 54, 55, 57, 58]. Experienced burden decreased slightly when care tasks could be shared [43, 45] and it helped keeping balance between work and care when the informal caregiver was employed [46]. For some informal caregivers, taking care of their relative gave them a sense of fulfillment [46]. They felt relieved, for instance when they managed the financial situation [47]. But sometimes these shared care tasks also caused tension between family members because of disagreements with decisions, less time to offer help or different ideas about care [46, 47, 54].

Informal caregivers also arranged help from outside the family system [45, 46, 48, 50]. They proactively established a network of professionals and services so that help would be easily accessible right from the start of caregiving [45, 48, 52, 58]. Living near or in urban areas was a helpful resource to navigate through the possibilities of support [55]. Informal caregivers involved formal support services in the caregiving process because the unpredictable illness trajectory was identified as challenging and they did not know what to expect from the future [46, 52]. General help services such as homecare nurses, general practitioners, physiotherapists or volunteers supported the informal caregivers (with caregiving tasks) at home [45–48, 50, 52, 54–56, 59, 61]. However, one study noted that community nursing networks provided support for severely ill persons and informal caregivers, but sometimes offered only limited hands-on care [61].

When the severely ill persons’ health status deteriorated, informal caregivers and their care recipients tried to get in touch with palliative care and hospice services. The access to palliative and hospice care services was a vital resource when caring for the severely ill person at the EOL [48, 55, 56]. However, some severely ill persons and informal caregivers reported difficulties in receiving these services due to a lack of referral [61].

Sometimes informal caregivers were in need of help to coordinate care between clinical teams, specialties, institution and delivery systems. Some of them also experienced additional costs and frustration due to scheduling timely primary care [60]. Specialist palliative care nurses provided crisis and liaison support by coordinating care among providers [52, 61]. To improve the caring situation at home, some informal caregivers used respite care [46, 48]. If the care at home was not possible anymore, severely ill persons moved to care facilities. Severely ill persons and their informal caregivers perceived inpatient settings such as hospitals or hospices as safe shelters and felt safe due to the continuous care they received [48, 56]. Some informal caregivers felt guilty when moving the care recipient to a care facility because they did not want the severely ill person to live and die in a new environment [45, 54]. However, while the transition period felt burdensome for informal caregivers [45], their burden decreased once the severely ill person was there [54]. Some severely ill persons also wished to enter a care facility when dying [54].

### Interplay between economic and social resources

Overall, economic resources – whether personal savings and assets, workplace support, or state-provided financial aid – interact closely with different forms of social support [24]. Intertwined social and economic resources can jointly support both the practical management of EOL care and the psychological well-being of severely ill persons and their informal caregivers. Economic resources (e.g., through affordable portable oxygen tanks) can influence social participation (emotional support) [44] and the use of formal support services (instrumental support) [9, 61]. Furthermore, social resources can influence the availability and accessibility of economic resources. For example, having a strong social network may enable individuals to borrow money to meet care-related needs – a form of instrumental support [9]. The need for appraisal support emerges in the context of feelings of shame or uncertainty about using external financial resources [47]. Furthermore, informational support enables informal caregivers and care recipients to understand and navigate legal needs, subsidies or workplace policies effectively [9, 45–47, 52, 61]. Finally, emotional support from employers and colleagues – through understanding, listening, and being receptive to caregivers’ needs – facilitates the reconciliation of work and caregiving duties, helping caregivers cope with stress while managing their responsibilities [9, 45, 46, 54, 57, 59].

## Discussion

Our review offers a comprehensive overview of the relevance and consequences of economic and social resources on alleviating the multifaceted burdens encountered by both severely ill persons and their informal caregivers at the EOL. The major themes included savings and assets, workplace resources for informal caregivers and state support and insurance as well as emotional, appraisal, informational and instrumental support.

All of the included studies in this review, despite being conducted in high-income countries with different healthcare and insurance policy frameworks, consistently emphasized the crucial role of economic resources in shaping EOL experiences. Although government and council support helped to cover some care-related costs at home, informal caregivers often faced financial hardship due to insufficient subsidies. These findings align with international evidence indicating that, despite varying healthcare and insurance frameworks across high-income countries, informal caregivers in EOL care frequently face significant financial and emotional burdens due to insufficient support systems. EOL care is associated with high costs due to the combination of intensive medical treatments, frequent use of healthcare services, additional private and informal caregiving, and incomplete coverage by public or private insurance, which together place substantial financial burdens on both families and public budgets. Coupled with intense emotional stress and the urgent need for workplace flexibility, these factors make caregiving at the EOL more immediate and pressing than in non-terminal illnesses, where demands are generally ongoing but less acute [62]. Additionally, these EOL-specific burdens are likely shaped by the sociocultural contexts, as considerable differences exist in caregiving practices, resource availability, and family involvement across different sociocultural settings. Cultural factors have a significant influence on expectations of care, care decisions, and preferences at the EOL [63]. Studies show that in lower-income countries, for example, caregiving tasks are more often taken on by younger female family members living in the same household, and informal support is often the only care option available [64]. In comparison, caregivers in high-income countries are older, often do not live in the same household, and are more likely to be professional or formally supported caregivers [65], as welfare state structures in high-income countries often provide additional formal support and financial relief [66].

Nevertheless, despite the availability of additional financial support in high-income countries, feelings of shame and misconceptions regarding eligibility for government financial support posed additional barriers, preventing some from seeking the necessary assistance [47, 48, 54, 57]. These findings are in line with those of Gladstone et al. [67] who further demonstrate that shame is not only a consequence of financial hardship but also a powerful driver that perpetuates it. Their research shows that people who feel ashamed about their financial situation tend to withdraw from managing their finances and avoid seeking help, which leads to poorer financial decisions and worsening hardship over time. They also elaborate that shame is a stronger motivator for avoidance than guilt, making people less likely to seek out support even when they need it.

Furthermore, while access to direct financial means such as assets or governmental funding was found to be important [9, 48, 54, 57], a particularly important economic resource was the capacity of individuals to influence and negotiate their own working conditions. This emphasizes that economic resources should not be understood solely in material terms, but also as factors that affect autonomy and agency in the workplace and in the caregiving situation. These findings align with broader theoretical frameworks, such as Bourdieu’s Theory of Capital [68], that conceptualizes resources not only as tangible assets, but also as structurally mediated capabilities that determine the scope of action available to individuals within institutional hierarchies. Bourdieu distinguishes between several types of capital (economic, social, cultural, and symbolic), and emphasizes the interconnections between these types. With Bourdieu’s theory of capital in mind, it is important to recognize that the economic and social resources we focused on in our review can be understood as distinct yet intertwined forms of capital. Economic resources can facilitate access to social networks or help individuals maintain their positions within these networks, thus influencing their ability to leverage social capital [69]. Similarly, social capital (e.g., supportive relationships, networking opportunities) can enable individuals to better use economic resources, creating a synergistic effect that enhances their overall agency and well-being [70].

Our results highlight the significant role of relatives as informal caregivers – a particularly tangible expression of social capital – in easing the suffering of severely ill persons by providing various forms of social support. Informal caregivers’ essential contributions extended beyond providing practical assistance or meeting severely ill persons’ informational needs; they were particularly essential in offering emotional support [48, 50, 54]. In the scoping review by Schutter et al. [71], existing relationships such as those with family members, friends, and neighbors were also identified as the most important sources of social support for severely ill persons, family caregivers, and bereaved relatives.

In summary, we found that the lack of social and/or economic resources in EOL care can trigger burdens across physical, psychological, social, and spiritual dimensions. These findings are consistent with earlier studies [13, 72] highlighting the relevance of social and economic resources within the concept of “total pain” [15]. Although the concept was originally developed to capture and address the complex and holistic nature of suffering experienced by severely ill persons at the EOL, in line with recent research, our review underscores that informal caregivers were also exposed to various forms of burden [73]. While they often share the emotional distress of the severely ill person, they themselves are also subject to considerable strain due to the extensive caregiving responsibilities they are required to provide. They reported physical exhaustion related to caregiving tasks, psychological strain such as anxiety, experiences of social isolation, and spiritual crises [45, 48, 50, 54, 59]. Similar to the findings of Schutter et al. [71], we observed that informal caregivers themselves have significant needs for social support [9, 45, 46, 48–50, 54–59]. However, the need for social support, especially by friends or other like-minded people, may conflict with the phenomenon of the shrinking social world where informal caregivers experience increasing social isolation due to the severely ill persons deteriorating health and the caregiving situation, reducing time available for social contacts [48]. Additionally, Hajek et al. [74] demonstrate that informal caregivers’ isolation may be partly due to the social environment’s inability to comprehend the caregiving situation, leading to a lack of understanding and support. This may explain the importance of exchange with fellow sufferers mentioned by informal caregivers in the included studies [45, 48, 57]. Peer support may therefore play a crucial role in alleviating isolation [75] and reveals promising evidence for improving informal caregivers’ psychological health [76]. Building on this, the results of our review indicate the importance of coordinated efforts among health and social care professionals, non-governmental organizations, and volunteer services. For health professionals, the findings emphasize continuity in the care process, shared decision-making, and open communication about financial hardship to reduce shame and foster trust. Social workers play a central role in alleviating bureaucratic burdens; by guiding families through complex administrative processes and addressing concerns about financial dependency, they help restore informal caregivers’ sense of control. The findings also demonstrate the critical role of employers, whose understanding and flexibility directly affect caregivers’ well-being. Community and volunteer organizations can counteract loneliness and isolation through companionship, provide practical assistance, and create local care networks. Each of these stakeholders along with policy makers contributes to addressing the complex social and economic needs of severely ill persons and their informal caregivers and form the foundation of a more compassionate approach to the EOL.

### Strengths and limitations

This systematic review provides a valuable contribution to understanding the relevance and consequences of economic and social resources for seriously ill persons at the EOL as well as their informal caregivers by systematically synthesizing and integrating existing empirical evidence. It was conducted in accordance with established methodological standards, following the PRISMA guideline and therefore, ensuring transparency and reproducibility.

We included studies without a time limit, which allowed for a broad range of perspectives but introduced variation regarding time periods. Changes in economic conditions or healthcare systems over the years may have influenced participants’ experiences and should be considered when interpreting the findings.

Furthermore, the included studies were conducted in various high-income countries with different healthcare systems and insurance structures. This enhances the generalizability of the findings across diverse cultural and societal contexts. However, regarding social resources, a comparison with low-income countries could have provided valuable insights into how different economic and cultural contexts shape the availability and significance of social support at the EOL.

A potential limitation was the broad range of possible search terms. We could not include all of the search terms initially considered, as this would likely have produced an unmanageably large number of results beyond the scope of our resources. However, we prioritized and selected search terms based on those used in relevant reviews in the field which also increased comparability.

## Conclusions

Severely ill persons and their relatives are confronted with substantial challenges in managing the multifaceted demands of EOL care. These demands encompass not only practical and administrative aspects but, more significantly, the emotional strain associated with the situation. The reviewed literature underscores that the capacity to navigate these challenges and consequently, the degree of burden experienced by severely ill persons and their informal caregivers is clearly influenced by the availability of economic and social resources.

Particular attention should be given to informal caregivers, who often function as both providers and recipients of social support in EOL care. While they provide essential emotional and practical assistance to severely ill persons, they simultaneously rely on support themselves to manage the considerable psychological and physical demands placed upon them. Given the critical role of social support in alleviating the burden on informal caregivers, further research should prioritize the development and evaluation of both professional and peer support interventions to enhance informal caregivers’ well-being and coping strategies. These interventions should also address the ongoing needs of informal caregivers offering continuous emotional and practical support as they transition into aftercare.

The literature further highlights that balancing employment and caregiving poses a significant challenge for informal caregivers. To better support this balance, both workplace-level initiatives by employers and policy-level changes are necessary. One potential measure could be the introduction of income replacement schemes, similar to those available for parental leave.

Demographic changes are expected to lead to an increase in the number of informal caregivers, as the growing demand for care cannot be fully met by professional healthcare personnel alone. In this context, measures that improve the compatibility of employment and caregiving, as well as the promotion of so-called Compassionate Communities, gain importance. Compassionate Communities facilitate mutual support during times of serious illness, crises, and EOL. They are based on a public health approach that emphasizes shared societal responsibility for care and support, rather than viewing this as the sole duty of formal health and social care systems.

Within this approach, communities are regarded as equal partners in the design and delivery of high-quality EOL care. However, the development of such communities requires appropriate frameworks and political support to ensure their sustainable establishment. Future research should particularly investigate how Compassionate Communities can enhance the well-being of informal caregivers – such as by reducing psychological burden, social isolation, and caregiver overload. Furthermore, it would be useful to explore the concrete benefits these approaches offer in the context of palliative care, especially regarding the quality, continuity, and accessibility of care. In the long term, it is also important to evaluate how Compassionate Communities influence societal attitudes toward illness, dying, and bereavement, as well as their potential economic impacts, for example, by alleviating pressure on formal healthcare systems.

## Supplementary Information

Supplementary Material 1. PRISMA checklist.

Supplementary Material 2. Search strategy.

Supplementary Material 3. Critical appraisal.

## Data Availability

All data analyzed are included in published studies that were identified through databases as mentioned in the methods section. For further information, please contact the corresponding author.

## Abbreviations

EOL: End of life
JBI: Joanna Briggs Institute
