# Supplementary material for: Relevance and consequence of economic and social resources of severely ill persons and their informal caregivers at the end-of-life: a systematic review of qualitative studies
Source: BMC Palliat Care. 2025 Dec 12;25:22. doi: 10.1186/s12904-025-01961-6 (PMC12817415; doi:10.1186/s12904-025-01961-6)
Supplement: Supplementary file 1 — Supplementary Material 1. PRISMA checklist. [file 12904_2025_1961_MOESM1_ESM.docx]

| **Section and Topic** | **Item #** | **Checklist item** | **Location where item is reported** |
| --- | --- | --- | --- |
| **TITLE** | | |  |
| Title | 1 | Identify the report as a systematic review. | p. 1 |
| **ABSTRACT** | | |  |
| Abstract | 2 | See the PRISMA 2020 for Abstracts checklist. | p. 2-3 |
| **INTRODUCTION** | | |  |
| Rationale | 3 | Describe the rationale for the review in the context of existing knowledge. | p. 4-5, background |
| Objectives | 4 | Provide an explicit statement of the objective(s) or question(s) the review addresses.  *The aim of this review is to explore and understand the perceptions of severely ill persons and their informal caregivers regarding the relevance and consequences of their economic and social resources at the EOL in varying settings of high-income countries. Gaining a deep understanding of these factors is essential for developing targeted support strategies that address the holistic needs of severely ill persons at the EOL and their informal caregivers.* | p. 5-6, background |
| **METHODS** | | |  |
| Eligibility criteria | 5 | Specify the inclusion and exclusion criteria for the review and how studies were grouped for the syntheses.  *Population: Severely ill persons and/or their (bereaved) informal caregivers at the EOL*  *Phenomenon of Interest: Articles which described perspectives and perceptions around economic and/or social resources that were relevant for the care situation at the EOL of severely ill persons*  *Context: Research that was conducted in high income countries, as defined by World Bank List of Economies 2025* | p. 6-8, eligibility criteria |
| Information sources | 6 | Specify all databases, registers, websites, organisations, reference lists and other sources searched or consulted to identify studies. Specify the date when each source was last searched or consulted.  *We searched MEDLINE via PubMed, CINAHL and PsychInfo and did a backward search by screening reference lists of relevant studies. The last search was on October, 15^th^, 2025.* | p. 8, information sources |
| Search strategy | 7 | Present the full search strategies for all databases, registers and websites, including any filters and limits used.  *The search terms were developed, refined and agreed between 4 authors. We used the components “end of life”, “economic resources”, “social resources” and “qualitative research” and identified relevant key words and medical subject headings, also from existing literature reviews. We adapted the search string to each database’s specific syntax.* | p. 8-9, search strategy  + supplementary file 2 |
| Selection process | 8 | Specify the methods used to decide whether a study met the inclusion criteria of the review, including how many reviewers screened each record and each report retrieved, whether they worked independently, and if applicable, details of automation tools used in the process.  *First, we exported the search results from the individual databases into the citation management program Citavi software (version 7), where duplicate records were identified and removed. After de-duplication, we uploaded the remaining results to the review management platform RAYYAN. Four authors conducted the screening* *of title and abstract independently. We then retrieved the full texts for studies deemed eligible and reviewed them independently according to the eligibility criteria. In case of discrepancy or uncertainty, papers were read by a fifth author, discussed in the team and a final decision made.* | p. 9 selection process |
| Data collection process | 9 | Specify the methods used to collect data from reports, including how many reviewers collected data from each report, whether they worked independently, any processes for obtaining or confirming data from study investigators, and if applicable, details of automation tools used in the process.  *We developed data extraction forms. Three author extracted data using Microsoft Excel. We verified the extracted data through mutual double checks.* | p. 9 data collection process |
| Data items | 10a | List and define all outcomes for which data were sought. Specify whether all results that were compatible with each outcome domain in each study were sought (e.g. for all measures, time points, analyses), and if not, the methods used to decide which results to collect.  *We extracted all qualitative findings related to economic and social resources for severely ill persons at the end of life and their informal caregivers.* | p. 9 data collection process, p. 16-33 Table 4 summary of included studies |
|  | 10b | List and define all other variables for which data were sought (e.g. participant and intervention characteristics, funding sources). Describe any assumptions made about any missing or unclear information.  *Additional data collected included first author, year of publication, setting, country, sample, type of illness, study design and study method. Where information was missing, we recorded this as "no data provided".* | p. 9 data collection process, p. 16-33 Table 4 summary of included studies |
| Study risk of bias assessment | 11 | Specify the methods used to assess risk of bias in the included studies, including details of the tool(s) used, how many reviewers assessed each study and whether they worked independently, and if applicable, details of automation tools used in the process.  *Four authors rated the methodological quality of the included studies using the JBI Critical Appraisal Checklist for Qualitative Research. We verified the ratings through mutual double checks. Any discrepancies were resolved through discussion with EB.* *We did not exclude any articles from this literature review due to poor methodological quality.* | p.10, critical appraisal |
| Effect measures | 12 | Specify for each outcome the effect measure(s) (e.g. risk ratio, mean difference) used in the synthesis or presentation of results. | not applicable, systematic review of qualitative studies |
| Synthesis methods | 13a | Describe the processes used to decide which studies were eligible for each synthesis (e.g. tabulating the study intervention characteristics and comparing against the planned groups for each synthesis (item #5)).  *We developed a table summarizing the characteristics of each included study (authors, year of publication, aim, setting, country, sample, type of illness, study design, study method, main findings). Based on this, we assessed which studies addressed the predefined research question and fit the inclusion criteria for the synthesis. Only studies providing empirical qualitative data relevant to the thematic categories were included.* | p. 16-33 Table 4, summary of included studies |
|  | 13b | Describe any methods required to prepare the data for presentation or synthesis, such as handling of missing summary statistics, or data conversions.  *We extracted relevant findings (e.g. coded categories, participant quotes) from the results sections of each study. To ensure comparability, we reformulated categories where necessary without changing their meaning. No data conversions were required.* | p. 10-11, data analysis |
|  | 13c | Describe any methods used to tabulate or visually display results of individual studies and syntheses.  *We used summary tables to display key characteristics and main findings of each study. The extracted data were organized thematically.* | p. 16-33 Table 4, summary of included studies |
|  | 13d | Describe any methods used to synthesize results and provide a rationale for the choice(s). If meta-analysis was performed, describe the model(s), method(s) to identify the presence and extent of statistical heterogeneity, and software package(s) used.  *We analyzed the data using qualitative content analysis according to Schreier supported by MAXQDA software (version 2024). In a first step, we deductively developed overarching categories ‘economic resources’ and ‘social resources’. We further applied House’s framework of social support to deductively categorize social support into four distinct types, emerging from both natural (e.g. family, friends) and formal support systems (e.g. health professionals).* *This method was chosen because it allows for systematic and transparent structuring of complex qualitative data.* | p. 10-11, data analysis |
|  | 13e | Describe any methods used to explore possible causes of heterogeneity among study results (e.g. subgroup analysis, meta-regression). | not applicable, systematic review of qualitative studies |
|  | 13f | Describe any sensitivity analyses conducted to assess robustness of the synthesized results. | not applicable, systematic review of qualitative studies |
| Reporting bias assessment | 14 | Describe any methods used to assess risk of bias due to missing results in a synthesis (arising from reporting biases). | not applicable, systematic review of qualitative studies |
| Certainty assessment | 15 | Describe any methods used to assess certainty (or confidence) in the body of evidence for an outcome. | not applicable, systematic review of qualitative studies |
| **RESULTS** | | |  |
| Study selection | 16a | Describe the results of the search and selection process, from the number of records identified in the search to the number of studies included in the review, ideally using a flow diagram.  *In total, we identified 2,388 studies in the initial search with an additional 400 new records from the search from September 2024 to October 2025. After the removal of duplicates, we screened 1,550 studies in 2024 and 305 in the search update 2025. Initial screening of titles and abstracts resulted in 145 eligible papers for full-text review in 2024 and 30 records in 2025. A total of 13 studies from the initial search and 7 studies from the search update were included in the review. Overall, this review includes 20 studies.* | p. 11, results + prisma flow diagram |
|  | 16b | Cite studies that might appear to meet the inclusion criteria, but which were excluded, and explain why they were excluded.  *Studies were excluded because they did not meet the inclusion criteria, for example regarding the population (children instead of adults, different definition of end-of-life).* | p. 6-8, eligibility criteria + Table 1 eligibility criteria |
| Study characteristics | 17 | Cite each included study and present its characteristics. | p. 11-33, results + Table 4 summary of included studies |
| Risk of bias in studies | 18 | Present assessments of risk of bias for each included study.  *In all studies, the methodological approaches were transparently reported, allowing for a clear understanding and replication of the results but only seven studies provided information on the researchers’ cultural and theoretical stance, while also reflecting on the reciprocal influence between the investigators and the study outcomes.* | p. 13-15 quality appraisal + Table 3 quality appraisal synthesis |
| Results of individual studies | 19 | For all outcomes, present, for each study: (a) summary statistics for each group (where appropriate) and (b) an effect estimate and its precision (e.g. confidence/credible interval), ideally using structured tables or plots. | not applicable, systematic review of qualitative studies |
| Results of syntheses | 20a | For each synthesis, briefly summarise the characteristics and risk of bias among contributing studies. | No risk of bias assessment as inclusion of qualitative studies only, critical appraisal of each study is included, see above |
|  | 20b | Present results of all statistical syntheses conducted. If meta-analysis was done, present for each the summary estimate and its precision (e.g. confidence/credible interval) and measures of statistical heterogeneity. If comparing groups, describe the direction of the effect. | not applicable, systematic review of qualitative studies |
|  | 20c | Present results of all investigations of possible causes of heterogeneity among study results. | not applicable, systematic review of qualitative studies |
|  | 20d | Present results of all sensitivity analyses conducted to assess the robustness of the synthesized results. | not applicable, systematic review of qualitative studies |
| Reporting biases | 21 | Present assessments of risk of bias due to missing results (arising from reporting biases) for each synthesis assessed. | not applicable, systematic review of qualitative studies |
| Certainty of evidence | 22 | Present assessments of certainty (or confidence) in the body of evidence for each outcome assessed. | not applicable, systematic review of qualitative studies |
| **DISCUSSION** | | |  |
| Discussion | 23a | Provide a general interpretation of the results in the context of other evidence. | p. 45-49, discussion |
|  | 23b | Discuss any limitations of the evidence included in the review. | p. 49-50, strengths and limitations |
|  | 23c | Discuss any limitations of the review processes used. | p. 49-50, strengths and limitations |
|  | 23d | Discuss implications of the results for practice, policy, and future research. | p. 50-51, conclusions |
| **OTHER INFORMATION** | | |  |
| Registration and protocol | 24a | Provide registration information for the review, including register name and registration number, or state that the review was not registered. | not applicable, no registration |
|  | 24b | Indicate where the review protocol can be accessed, or state that a protocol was not prepared. | not applicable, protocol not prepared |
|  | 24c | Describe and explain any amendments to information provided at registration or in the protocol. | not applicable |
| Support | 25 | Describe sources of financial or non-financial support for the review, and the role of the funders or sponsors in the review.  *The research project is made possible by a research grant from the AGE-Stiftung.* | p. 52, funding |
| Competing interests | 26 | Declare any competing interests of review authors.  *The authors declare that they have no competing interests.* | p. 53, competing interests |
| Availability of data, code and other materials | 27 | Report which of the following are publicly available and where they can be found: template data collection forms; data extracted from included studies; data used for all analyses; analytic code; any other materials used in the review.  *All data analyzed are included in published studies that were identified through databases as mentioned in the methods section. For further information, please contact the corresponding author.* | p. 52, availability of data and materials |

*From:*  Page MJ, McKenzie JE, Bossuyt PM, Boutron I, Hoffmann TC, Mulrow CD, et al. The PRISMA 2020 statement: an updated guideline for reporting systematic reviews. BMJ 2021;372:n71. doi: 10.1136/bmj.n71. This work is licensed under CC BY 4.0. To view a copy of this license, visit <https://creativecommons.org/licenses/by/4.0/>
