# Supplementary material for: Relevance and consequence of economic and social resources of severely ill persons and their informal caregivers at the end-of-life: a systematic review of qualitative studies
Source: BMC Palliat Care. 2025 Dec 12;25:22. doi: 10.1186/s12904-025-01961-6 (PMC12817415; doi:10.1186/s12904-025-01961-6)
Supplement: Supplementary file 2 — Supplementary Material 2. Search strategy. [file 12904_2025_1961_MOESM2_ESM.docx]

Supplementary File 2

**PubMed**

"Qualitative Research"[Mesh] OR (Interview*[tiab])) OR (focus group*[tiab])) OR (qualitative*[tiab]))

AND

Socioeconomic*[tiab]) OR ("socio-economic*"[tiab])) OR (neighbor*[tiab])) OR (neighbour*[tiab])) OR (relativ*[tiab])) OR (famil*[tiab])) OR (friend*[tiab])) OR ("social class*"[tiab])) OR ("social support"[tiab])) OR ("Social Support"[Mesh])) OR ("Social Capital"[Mesh])) OR ("social capital"[tiab])) OR ("social resource*"[tiab])))

AND

((((((((((((((((((("end-stage"[tiab]) OR ("Terminal*"[tiab])) OR (Dying[tiab])) OR ("Advanced illness*"[tiab])) OR ("Life limit*"[tiab])) OR (EOL[tiab])) OR ("Last year of life"[tiab])) OR (EOLC[tiab]))) OR ("Advanced cancer"[tiab])) OR ("Advanced progressive illness"[tiab])) OR (hospice*[tiab])) OR ("end of life"[tiab])) OR ("hospices"[Mesh])) OR (palliat*[tiab])) OR ("palliative medicine"[Mesh])) OR ("terminal care"[Mesh])) OR ("Palliative Care"[Mesh])) OR (("Hospice Care"[Mesh]))))

AND

(((((((((((((((savings[tiab]) OR (salar*[tiab])) OR (wage*[tiab])) OR (income[tiab])) OR (job[tiab])) OR (employ*[tiab])) OR (Occupation[tiab])) OR ("financial toxicity"[tiab])) OR ("economic burden*"[tiab])) OR ("financial burden*"[tiab])) OR ("financial hardship*"[tiab])) OR ("financial resource*"[tiab])) OR ("economic resource*"[tiab])) OR (financial stress[MeSH Major Topic])) OR ("financial strain"[tiab]))

**PsychInfo**

"end-stage".ti. or "end-stage".ab. or terminal*.ti. or terminal*.ab. or dying.ti. or dying.ab. or "Advanced illness*".ti. or "Advanced illness*".ab. or "Life limit*".ti. or "Life limit*".ab or EOL.ti. or EOL.ab. or "Last year of life".ti. or "Last year of life".ab. OR EOLC.ti. or EOLC.ab. "Advanced cancer".ti. or "Advanced cancer".ab. or "Advanced progressive illness".ab. or "Advanced progressive illness".ti. or hospice*.ab. or hospice*.ti.or "end of life".ab. or "end of life".ti. or palliat*.ab. or palliat*.ti. or exp hospice/ or exp palliative care/ or exp terminally ill patients/ or exp terminal cancer/ or exp "death and dying"/

AND exp social support/ OR exp social capital/ OR Socioeconomic*.ab. or Socioeconomic*.ti. OR "socio-economic*".ab. or "socio-economic*".ti. OR neighbor*.ab. or neighbor*.ti. OR neighbour*.ab. or neighbour*.ti. OR relativ*.ab. or relativ*.ti. OR famil*.ab. or famil*.ti. OR friend*.ab. or friend*.ti. "social class*".ab. or "social class*".ti. OR "social support".ab. or "social support".ti. OR "social capital".ab. or "social capital".ti. OR "social resource*".ab. or "social resource*".ti.

AND **savings.ab. OR savings.ti. OR salar*.ab. or salar*.ti. OR wage*.ab. or wage*.ti. OR income.ab. or income.ti. OR job.ab. or job.ti. OR employ*.ab. or employ*.ti. OR Occupation.ab. or Occupation.ti. OR "financial toxicity".ab. or "financial toxicity".ti. OR "economic burden*".ab. or "economic burden*".ti. OR "financial burden*".ab. or "financial burden*".ti. OR "financial hardship*".ab. OR "financial hardship*".ti. OR "financial strain".ti. or "financial strain".ab. OR "financial resource*".ab. or "financial resource*".ti. OR "economic resource*".ab. or "economic resource*".ti. OR exp financial strain/**

**AND** exp qualitative methods/ OR interview*.ti. or interview*.ab. OR "focus group*".ti. or "focus group*".ab. OR qualitative*.ti. or qualitative*.ab.

**CINAHL**

((((((((((((((((((TI “end-stage”) OR (AB “end-stage”)) OR (TI “Terminal*”) OR (AB “Terminal*”)) OR (TI “Dying”) OR (AB “Dying”)) OR (TI “Advanced illness*”) OR (AB “Advanced illness*”)) OR (TI “Life limit*”) OR (AB “Life limit*”)) OR (TI “EOL”) OR (AB “EOL”)) OR (TI “Last year of life”) OR (AB “Last year of life”)) OR (TI “EOLC”) OR (AB “EOLC”)) OR (TI “Advanced cancer”) OR (AB “Advanced cancer”)) OR (TI “Advanced progressive illness”) OR (AB “Advanced progressive illness”)) OR (TI “hospice*”) OR (AB “hospice*”)) OR (TI “end of life”) OR (AB “end of life”)) OR (MH “Hospices”)) OR (TI “palliat*”) OR (AB “palliat*”)) OR (MH “Palliative Medicine”)) OR (MH “Terminal Care+”)) OR (MH “Palliative Care”)) OR (MH “Hospice Care”))

**AND** ((((((((((((((TI “Socioeconomic*”) OR (AB “Socioeconomic*”)) OR (TI “socio-economic*”) OR (AB “socio-economic*”)) OR (TI “neighbor*”) OR (AB “neighbor*”)) OR (TI “neighbour*”) OR (AB “neighbour*”)) OR (TI “relativ*”) OR (AB “relativ*”)) OR (TI “famil*”) OR (AB “famil*”)) OR (TI “friend*”) OR (AB “friend*”)) OR (TI “social class*”) OR (AB “social class*”)) OR (TI “social support”) OR (AB “social support”)) OR (MH “Support, Social+”)) OR (MH “Social Capital”)) OR (TI “social capital”) OR (AB “social capital”)) OR (TI “social resource*”) OR (AB “social resource*”))

**AND** (((((((((((((((TI savings) OR (AB savings)) OR (TI salar*) OR (AB salar*)) OR (TI wage*) OR (AB wage*)) OR (TI income) OR (AB income)) OR (TI job) OR (AB job)) OR (TI employ*) OR (AB employ*)) OR (TI Occupation) OR (AB Occupation)) OR (TI “financial toxicity”) OR (AB “financial toxicity”)) OR (TI “economic burden*”) OR (AB “economic burden*”)) OR (TI “financial burden*”) OR (AB “financial burden*”)) OR (TI “financial hardship*”) OR (AB “financial hardship*”)) OR (TI “financial resource*”) OR (AB “financial resource*”)) OR (TI “economic resource*”) OR (AB “economic resource*”)) OR (TI “financial strain”) OR (AB “financial strain”)) OR (MH “Financial Stress”))

AND ((((TI Interview*) OR (AB Interview*)) OR (TI “focus group*”) OR (AB “focus group*”)) OR (TI qualitative*) OR (AB qualitative*)) OR (MH “Qualitative Studies+))
