# Supplementary material for: Relevance and consequence of economic and social resources of severely ill persons and their informal caregivers at the end-of-life: a systematic review of qualitative studies
Source: BMC Palliat Care. 2025 Dec 12;25:22. doi: 10.1186/s12904-025-01961-6 (PMC12817415; doi:10.1186/s12904-025-01961-6)
Supplement: Supplementary file 3 — Supplementary Material 3. Critical appraisal. [file 12904_2025_1961_MOESM3_ESM.docx]

Supplementary File 3

JBI Critical Appraisal Checklist for
Qualitative Research

Reviewer: Daniela Bernhardsgrütter Date: 06.11.2025

Author: Seng Hock Martin Ang, Wing Hong Edward Poon, Odette Best and Coralie Graham
Year: 2024
Record Number: 1

|  | Yes | No | Unclear | Not applicable |
| --- | --- | --- | --- | --- |
| 1. Is there congruity between the stated philosophical perspective and the research methodology?   The study adopts a comprehensive and interpretive philosophical perspective, which aligns with the hermeneutic phenomenological approach | □ | □ | □ | □ |
| 1. Is there congruity between the research methodology and the research question or objectives?   The hermeneutic phenomenological approach, which is well aligned with the aim to explore the lived experiences of family caregivers providing EOL cancer care. | □ | □ | □ | □ |
| 1. Is there congruity between the research methodology and the methods used to collect data?   The use of semi-structured in-depth interviews, field observations and a reflective journal aligns well with the research aim. | □ | □ | □ | □ |
| 1. Is there congruity between the research methodology and the representation and analysis of data?   Data analysis was based on van Manen’s six-step approach to hermeneutics phenomenology. Reflexivity was used to deeply engage with the textual data. | □ | □ | □ | □ |
| 1. Is there congruity between the research methodology and the interpretation of results?   The authors provided rich descriptions of themes and sub-themes, supported by participants’ quotations. | □ | □ | □ | □ |
| 1. Is there a statement locating the researcher culturally or theoretically?   The study clearly identifies the second author’s theoretical position, which informs and shapes his role in the independent coding and interpretation of the data. | □ | □ | □ | □ |
| 1. Is the influence of the researcher on the research, and vice- versa, addressed?   The interpretive findings were strengthened through  investigator triangulation. | □ | □ | □ | □ |
| 1. Are participants, and their voices, adequately represented?   Numerous direct quotations illustrate themes clearly and authentically. | □ | □ | □ | □ |
| 1. Is the research ethical according to current criteria or, for recent studies, and is there evidence of ethical approval by an appropriate body?   Yes. Ethics approval and informed consent described | □ | □ | □ | □ |
| 1. Do the conclusions drawn in the research report flow from the analysis, or interpretation, of the data?   Yes. Themes well-grounded in verbatim quotes; coherence between data and conclusions. | □ | □ | □ | □ |

Overall appraisal: Include □ Exclude □ Seek further info □

**Comments (Including reason for exclusion):**

This study was thoroughly conducted and described. All aspects were elaborated in detail and can be evaluated positively.

JBI Critical Appraisal Checklist for
Qualitative Research

Reviewer: Fabiola Orosaj Date: 28.01.2025

Author: Femmy M. Bjinsdorp, Bregje D. Onwuteaka-Philipsen, Cécile R.L. Boot, Allard J. van der Beek, Hanna T. Klop and H. Roeline W. Pasman
Year: 2021
Record Number: 2

|  | Yes | No | Unclear | Not applicable |
| --- | --- | --- | --- | --- |
| 1. Is there congruity between the stated philosophical perspective and the research methodology?   The study is a longitudinal qualitative study with in-depth interviews. The study adopts a qualitative research methodology, employing thematic analysis, which is well-suited for exploring the subjective experiences of family caregivers. This approach aligns with an interpretative research perspective. | □ | □ | □ | □ |
| 1. Is there congruity between the research methodology and the research question or objectives?   The research question fits the method because it focuses on the subjective experiences and needs of family caregivers, making a qualitative approach with interviews appropriate. | □ | □ | □ | □ |
| 1. Is there congruity between the research methodology and the methods used to collect data?   Semi structured, in-depth interviews were held. This is appropriate for qualitative research to assess subjective experiences. | □ | □ | □ | □ |
| 1. Is there congruity between the research methodology and the representation and analysis of data?   All interviews were transcribed verbatim and were analyzed following the principles of thematic  analysis. This is appropriate for qualitative research to assess subjective experiences. | □ | □ | □ | □ |
| 1. Is there congruity between the research methodology and the interpretation of results?   The family caregivers’ experiences were summed up in four domains, namely characteristics of the caregiver, the care situation, the situation at work and the context. The interpretations of the researcher were checked by the participant after summarizing the most important themes during and at the end of the interview. This is appropriate for qualitative research to assess subjective experiences. | □ | □ | □ | □ |
| 1. Is there a statement locating the researcher culturally or theoretically?   There is no statement locating the researcher culturally or theoretically. But all interviews were conducted by one female researcher who was trained in in-depth interviewing. | □ | □ | □ | □ |
| 1. Is the influence of the researcher on the research, and vice- versa, addressed?   There is no statement according to the influence of the researcher on the research and vice-versa. | □ | □ | □ | □ |
| 1. Are participants, and their voices, adequately represented?   There are quotes for all domains. | □ | □ | □ | □ |
| 1. Is the research ethical according to current criteria or, for recent studies, and is there evidence of ethical approval by an appropriate body?   The study protocol was approved by the Medical Ethics Review Committee of VU University Medical Center (reference 2018.113). Participants provided written informed consent prior to the interview. Transcripts were anonymized to ensure privacy. | □ | □ | □ | □ |
| 1. Do the conclusions drawn in the research report flow from the analysis, or interpretation, of the data?   Conclusions are based on findings from interviews and analysis. | □ | □ | □ | □ |

Overall appraisal: Include □ Exclude □ Seek further info □

Comments (Including reason for exclusion)

In principle, the study meets the essential requirements. However, two aspects (points 6 and 7) remain unclear: the researchers did not provide any statements regarding their beliefs and values or how these may have influenced the study. Furthermore, it is unclear how the relationship between the researchers and the study participants was addressed.

JBI Critical Appraisal Checklist for
Qualitative Research

Reviewer: Fabiola Orosaj Date: 28.01.2025

Author: Femmy M. Bjinsdorp, Bregje D. Onwuteaka-Philipsen, Cécile R.L. Boot, Allard J. van der Beek and H. Roeline W. Pasman
Year: 2022
Record Number: 3

|  | Yes | No | Unclear | Not applicable |
| --- | --- | --- | --- | --- |
| 1. Is there congruity between the stated philosophical perspective and the research methodology?   The study is a longitudinal qualitative study with in-depth interviews. The study adopts a qualitative research methodology, employing thematic analysis, which is well-suited for exploring the subjective experiences of family caregivers. This approach aligns with an interpretative research perspective | □ | □ | □ | □ |
| 1. Is there congruity between the research methodology and the research question or objectives?   The research question fits the method because it focuses on the subjective experiences and needs of family caregivers, making a qualitative approach with interviews appropriate. | □ | □ | □ | □ |
| 1. Is there congruity between the research methodology and the methods used to collect data?   Semi structured, in-depth interviews were held. This is appropriate for qualitative research to assess subjective experiences. | □ | □ | □ | □ |
| 1. Is there congruity between the research methodology and the representation and analysis of data?   The longitudinal analysis used in the current paper was based on 53 interviews, carried out with 17 participants over a time period of 2.5 years. All transcripts were analyzed as a single unit. Individual trajectories were then drawn in a graph with separate lines indicating changes over time. This is appropriate for qualitative research. | □ | □ | □ | □ |
| 1. Is there congruity between the research methodology and the interpretation of results?   Qualitative analysis revealed that all family caregivers  experienced a burden to some extent over the course of the illness trajectory. Broadly, two groups were identified, each with a similar course in the level of caregiver burden over time. | □ | □ | □ | □ |
| 1. Is there a statement locating the researcher culturally or theoretically?   There is no statement locating the researcher culturally or theoretically. | □ | □ | □ | □ |
| 1. Is the influence of the researcher on the research, and vice- versa, addressed?   There is no statement according to the influence of the researcher on the research and vice-versa. | □ | □ | □ | □ |
| 1. Are participants, and their voices, adequately represented?   There are quotes for all trajectories. | □ | □ | □ | □ |
| 1. Is the research ethical according to current criteria or, for recent studies, and is there evidence of ethical approval by an appropriate body?   The Medical Ethics Review Committee of VU University Medical Center approved the study protocol (reference 2018.113). All methods were performed in accordance with the Declaration of Helsinki. Participants provided  written informed consent prior to their first interview. At the end of each interview, participants were asked if they could be contacted again for a subsequent interview. Transcripts were anonymized to ensure privacy. | □ | □ | □ | □ |
| 1. Do the conclusions drawn in the research report flow from the analysis, or interpretation, of the data?   Conclusions are based on findings from interviews and analysis. | □ | □ | □ | □ |

Overall appraisal: Include □ Exclude □ Seek further info □

Comments (Including reason for exclusion)

In principle, the study meets the essential requirements. However, two aspects (points 6 and 7) remain unclear: the researchers did not provide any statements regarding their beliefs and values or how these may have influenced the study. Furthermore, it is unclear how the relationship between the researchers and the study participants was addressed.

JBI Critical Appraisal Checklist for
Qualitative Research

Reviewer: Fabiola Orosaj Date: 28.01.2025

Author: Helen Close, Kamal Sidhu, Hazel Genn, Jonathan Ling and Colette Hawkings
Year: 2021
Record Number: 4

|  | Yes | No | Unclear | Not applicable |
| --- | --- | --- | --- | --- |
| 1. Is there congruity between the stated philosophical perspective and the research methodology?   The study is a qualitative study. The study adopts a qualitative research methodology, employing reflexive thematic analysis, which is well-suited for exploring the subjective experiences. This approach aligns with an interpretative research perspective. | □ | □ | □ | □ |
| 1. Is there congruity between the research methodology and the research question or objectives?   The research question fits the method because it focuses on the subjective experiences of patients at the end of life and their informal carers, making a qualitative approach with interviews appropriate. | □ | □ | □ | □ |
| 1. Is there congruity between the research methodology and the methods used to collect data?   A semi-structured interview guide was used, developed by a multiprofessional project steering group which included patient/carer representatives. The guide incorporated questions about current experience and plans for the future. This approach aligns with an interpretative research perspective. | □ | □ | □ | □ |
| 1. Is there congruity between the research methodology and the representation and analysis of data?   Data got analyzed using the six-phase approach to reflexive thematic analysis. This entailed familiarization with the data then initial coding through application of descriptive words to significant phrases. This approach aligns with an interpretative research perspective. | □ | □ | □ | □ |
| 1. Is there congruity between the research methodology and the interpretation of results?   The experiences were summed up in eight themes. This is appropriate for qualitative research to assess subjective experiences. | □ | □ | □ | □ |
| 1. Is there a statement locating the researcher culturally or theoretically?   At the end of the study there is a short acknowledgment about the researchers, but there is no further information or statement locating the researcher culturally or theoretically. | □ | □ | □ | □ |
| 1. Is the influence of the researcher on the research, and vice- versa, addressed?   There is no statement according to the influence of the researcher on the research and vice-versa. | □ | □ | □ | □ |
| 1. Are participants, and their voices, adequately represented?   There are quotes for all themes. | □ | □ | □ | □ |
| 1. Is the research ethical according to current criteria or, for recent studies, and is there evidence of ethical approval by an appropriate body?   Research ethics approval was granted by East Midlands (Nottingham 1) NHS Ethics Committee (REC: 18/EM/0021, February 2018). Health Research Authority approval was granted (IRAS ID 236775). Written informed consent was obtained from participants in the study. | □ | □ | □ | □ |
| 1. Do the conclusions drawn in the research report flow from the analysis, or interpretation, of the data?   Conclusions are based on findings from interviews and analysis. | □ | □ | □ | □ |

Overall appraisal: Include □ Exclude □ Seek further info □

Comments (Including reason for exclusion)

In principle, the study meets the essential requirements. However, two aspects (points 6 and 7) remain unclear: the researchers did not provide any statements regarding their beliefs and values or how these may have influenced the study. Furthermore, it is unclear how the relationship between the researchers and the study participants was addressed.

JBI Critical Appraisal Checklist for
Qualitative Research

Reviewer: Gerald Michelak Date: 15.01.2025

Author: Marjorie Coristine, Dauna Crooks, Eva Grunfeld, Carole Stonebridge and Anne Christie
Year: 2003
Record Number: 5

|  | Yes | No | Unclear | Not applicable |
| --- | --- | --- | --- | --- |
| 1. Is there congruity between the stated philosophical perspective and the research methodology?   The study adopts a qualitative, descriptive approach to understand the subjective experiences of caregivers, aligning well with a constructivist philosophical perspective. The use of focus groups and content analysis fits this epistemological stance. | □ | □ | □ | □ |
| 1. Is there congruity between the research methodology and the research question or objectives?   The research question aims to describe the psychosocial impact of caregiving, which justifies the use of qualitative methods such as focus groups and content analysis. The methodology is appropriate to capture the complex, personal experiences of participants. | □ | □ | □ | □ |
| 1. Is there congruity between the research methodology and the methods used to collect data?   Focus groups as a data collection method are typical for qualitative research and consistent with the chosen methodology. Open-ended questions and moderated group discussions allow for in-depth exploration of caregivers’ experiences. | □ | □ | □ | □ |
| 1. Is there congruity between the research methodology and the representation and analysis of data?   Data analysis was conducted through thematic coding and classification, which aligns with qualitative methodology. The presentation of results using categories and participant quotes reflects the chosen approach. | □ | □ | □ | □ |
| 1. Is there congruity between the research methodology and the interpretation of results?   The interpretation remains closely tied to the identified themes and participant statements, as it is common in qualitative studies. Conclusions are drawn directly from the analysed data without overinterpretation. | □ | □ | □ | □ |
| 1. Is there a statement locating the researcher culturally or theoretically?   Researchers are institutionally introduced, but there is no explicit cultural or theoretical self-location. The theoretical framework is not explicitly reflected upon. | □ | □ | □ | □ |
| 1. Is the influence of the researcher on the research, and vice- versa, addressed?   The study does not explicitly discuss the researchers’ influence on the research process or vice versa. There is no reflection on potential biases or the researchers’ roles. | □ | □ | □ | □ |
| 1. Are participants, and their voices, adequately represented?   Participants’ voices are well represented through numerous direct quotes and detailed descriptions of both spouse and non-spouse caregivers. The diversity of experiences is presented in a nuanced manner. | □ | □ | □ | □ |
| 1. Is the research ethical according to current criteria or, for recent studies, and is there evidence of ethical approval by an appropriate body?   The study explicitly states that ethical approval was obtained from relevant ethics boards. Informed consent was secured from participants for participation and use of quotes. | □ | □ | □ | □ |
| 1. Do the conclusions drawn in the research report flow from the analysis, or interpretation, of the data?   Conclusions logically follow from the data analysis and identified themes. There are no unwarranted generalizations; findings are discussed in a balanced and differentiated way. | □ | □ | □ | □ |

Overall appraisal: Include □ Exclude □ Seek further info □

**Comments (Including reason for exclusion):**

In principle, the study meets the essential requirements. However, two aspects (points 6 and 7) remain unclear: the researchers did not provide any statements regarding their beliefs and values or how these may have influenced the study. Furthermore, it is unclear how the relationship between the researchers and the study participants was addressed; while a research nurses-participant-relationship was mentioned, no further details were provided about this relationship or its potential impact.

JBI Critical Appraisal Checklist for
Qualitative Research

Reviewer: Marlene Matzinger Date: 30.10.2025

Author: Ellis C. Dillon, Meghan C. Martinez, Martina Li, Amandeep K. Mann-Grewal, Harold S. Luft, Su-Ying Liang, Natalia Colocci, Steve Lai and Manali Patel
Year: 2024
Record Number: 6

|  | Yes | No | Unclear | Not applicable |
| --- | --- | --- | --- | --- |
| 1. Is there congruity between the stated philosophical perspective and the research methodology? The article mentions a pragmatic realist approach, which aligns with the mixed methods methodology. | □ | □ | □ | □ |
| 1. Is there congruity between the research methodology and the research question or objectives? The study's methodology is coherently aligned with the research question: the combined mixed-methods design, consisting of quantitative data collection and qualitative analysis, allows for both a comprehensive representation of relevant care experiences and an in-depth analysis of systemic barriers and burdens in advanced cancer and therefore answers the research question. | □ | □ | □ | □ |
| 1. Is there congruity between the research methodology and the methods used to collect data? Yes, the quantitative component allows for the statistical capture of patient characteristics and care patterns, while the qualitative interviews provide an in-depth understanding of individual experiences and challenges within the fragmented healthcare system. The selection and implementation of data collection are thus consistent and well-aligned with the methodology. | □ | □ | □ | □ |
| 1. Is there congruity between the research methodology and the representation and analysis of data? The mixed-methods methodology is implemented consistently: quantitative data from surveys and electronic health records are presented using descriptive statistics, while qualitative interviews are analyzed through inductive thematic analysis. The analysis presents the results in a manner consistent with the chosen approach. | □ | □ | □ | □ |
| 1. Is there congruity between the research methodology and the interpretation of results? The methodology (mixed-methods) is applied consistently through to the interpretation of results: both quantitative and qualitative findings are initially presented separately according to their methodological logic and subsequently interpreted together. | □ | □ | □ | □ |
| 1. Is there a statement locating the researcher culturally or theoretically?   The study provides information about the team (roles: sociologist, health economist, oncologists, palliative care physician, trained qualitative researchers) and about weekly meetings to discuss the authors positions and influence. | □ | □ | □ | □ |
| 1. Is the influence of the researcher on the research, and vice- versa, addressed?   Yes, the team met weekly to discuss progress, concerns, and the potential influence of their own positions, histories, and experiences. | □ | □ | □ | □ |
| 1. Are participants, and their voices, adequately represented? Yes. The study presents patients and caregivers' experiences and quotations, ensuring that participants' voices are central to the findings. | □ | □ | □ | □ |
| 1. Is the research ethical according to current criteria or, for recent studies, and is there evidence of ethical approval by an appropriate body?   The project was reviewed by the Sutter Health Institutional Review Board and was determined not to be human subjects research, so formal signed consent was not required. Participants got an informed written or verbal description of the study and how data would be used before they agreed to participate. | □ | □ | □ | □ |
| 1. Do the conclusions drawn in the research report flow from the analysis, or interpretation, of the data?   In the discussion and conclusion, the authors address key findings from both the quantitative and qualitative analyses—such as the impacts of fragmentation, administrative, and financial burdens. | □ | □ | □ | □ |

Overall appraisal: Include □ Exclude □ Seek further info □

Comments (Including reason for exclusion:

This study was thoroughly conducted and described. All aspects were elaborated in detail and can be evaluated positively.

JBI Critical Appraisal Checklist for
Qualitative Research - DiSSERTATION

Reviewer: Gerald Michelak Date: 15.01.2025

Author: Joan-Anne Dunsmore-Dawson
Year: 2020
Record Number: 7

|  | Yes | No | Unclear | Not applicable |
| --- | --- | --- | --- | --- |
| 1. Is there congruity between the stated philosophical perspective and the research methodology?   Strong congruity exists between the phenomenological philosophical perspective and the longitudinal qualitative methodology. The study explicitly draws on phenomenological principles to explore caregivers' lived experiences over time. | □ | □ | □ | □ |
| 1. Is there congruity between the research methodology and the research question or objectives?   The longitudinal qualitative cross-sectional design aligns well with the aim to explore family caregivers' experiences over time. The methodology enables capturing how experiences evolve throughout the caregiving journey. | □ | □ | □ | □ |
| 1. Is there congruity between the research methodology and the methods used to collect data?   The semi-structured interviews conducted at multiple time points, supplemented by the Pictor technique, are consistent with phenomenological inquiry. These methods effectively capture the depth and complexity of caregivers' experiences. | □ | □ | □ | □ |
| 1. Is there congruity between the research methodology and the representation and analysis of data?   The thematic analysis approach with iterative analysis of interviews as stand-alone studies, followed by longitudinal analysis, is appropriate for phenomenological research. The template analysis method supports systematic examination of patterns. | □ | □ | □ | □ |
| 1. Is there congruity between the research methodology and the interpretation of results?   The interpretation focuses on identifying patterns of change and stability in caregivers' experiences, consistent with phenomenological principles. The essential themes emerge directly from participants' accounts. | □ | □ | □ | □ |
| 1. Is there a statement locating the researcher culturally or theoretically?   The researcher clearly positions herself through reflexive statements about her background as an ODP (Operation Department Practitioner), volunteer, and bereavement counsellor. Her motivations and personal experiences with caregiving are explicitly addressed. | □ | □ | □ | □ |
| 1. Is the influence of the researcher on the research, and vice- versa, addressed?   Reflexivity is thoroughly addressed throughout the study. The researcher reflects on her engagement with participants, her dual role as counsellor/researcher, and how her background influenced data collection and interpretation. | □ | □ | □ | □ |
| 1. Are participants, and their voices, adequately represented?   Participants' voices are extensively represented through direct quotes and individual profiles. The longitudinal design allows for capturing the evolving nature of their experiences across multiple interviews. | □ | □ | □ | □ |
| 1. Is the research ethical according to current criteria or, for recent studies, and is there evidence of ethical approval by an appropriate body?   The study demonstrates strong ethical governance with explicit discussion of consent processes, confidentiality, and data management. Formal ethical approval was obtained and documented. | □ | □ | □ | □ |
| 1. Do the conclusions drawn in the research report flow from the analysis, or interpretation, of the data?   The conclusions regarding practical and emotional support needs, the role of palliative care services, and financial inequalities directly emerge from the thematic analysis and longitudinal perspective of the data. | □ | □ | □ | □ |

Overall appraisal: Include □ Exclude □ Seek further info □

**Comments (Including reason for exclusion):**

This qualitative dissertation was thoroughly conducted and described. All aspects were elaborated in detail and can be evaluated positively.

JBI Critical Appraisal Checklist for
Qualitative Research

Reviewer: Gerald Michelak Date: 16.01.2025

Author: Gail Enyert and Mary E. Burman
Year: 1999
Record Number: 8

|  | Yes | No | Unclear | Not applicable |
| --- | --- | --- | --- | --- |
| 1. Is there congruity between the stated philosophical perspective and the research methodology?   The study adopts a constructivist perspective, focusing on caregivers' subjective experiences, which aligns with the qualitative, grounded theory and ethnographic methodology. The aim to explore meaning and self-transcendence fits this approach. | □ | □ | □ | □ |
| 1. Is there congruity between the research methodology and the research question or objectives?   The qualitative methodology is appropriate for the study’s aim to explore caregivers’ perceptions of well-being and self-transcendence. Open-ended exploration allows for deep understanding of these phenomena. | □ | □ | □ | □ |
| 1. Is there congruity between the research methodology and the methods used to collect data?   Unstructured interviews are well-suited to grounded theory and ethnography, enabling participants to freely share their caregiving experiences. This method supports the study’s exploratory nature. | □ | □ | □ | □ |
| 1. Is there congruity between the research methodology and the representation and analysis of data?   Data were analyzed using grounded theory, with systematic coding and theme development, which matches the qualitative design. Themes and categories are clearly described and illustrated with participant quotes. | □ | □ | □ | □ |
| 1. Is there congruity between the research methodology and the interpretation of results?   Interpretation is closely tied to the emergent themes of meaning and self-transcendence, and conclusions are drawn directly from the data. The analysis remains grounded in participants’ accounts. | □ | □ | □ | □ |
| 1. Is there a statement locating the researcher culturally or theoretically?   The researchers’ professional roles are stated. | □ | □ | □ | □ |
| 1. Is the influence of the researcher on the research, and vice- versa, addressed?   Some steps were taken to check for researcher effects but the researchers’ potential influence on data collection and analysis is not deeply explored. | □ | □ | □ | □ |
| 1. Are participants, and their voices, adequately represented?   Participants’ voices are well represented through direct quotes and detailed narrative accounts. The variety of caregiving situations is described, giving insight into individual experiences. | □ | □ | □ | □ |
| 1. Is the research ethical according to current criteria or, for recent studies, and is there evidence of ethical approval by an appropriate body?   Ethical approval was obtained from the university’s review board, and informed consent was collected from all participants. Confidentiality and participant welfare were addressed. | □ | □ | □ | □ |
| 1. Do the conclusions drawn in the research report flow from the analysis, or interpretation, of the data?   The study’s conclusions about meaning, self-transcendence, and emotional well-being clearly arise from the data analysis. The findings are supported by participants’ narratives and thematic structure. | □ | □ | □ | □ |

Overall appraisal: Include □ Exclude □ Seek further info □

**Comments (Including reason for exclusion):**

In principle, the study meets the essential requirements. However, it is unclear how the relationship between the researchers and the study participants was addressed. Only the use of a research diary and reflections within the research team were described.

JBI Critical Appraisal Checklist for
Qualitative Research

Reviewer: Gerald Michelak Date: 16.01.2025

Authors: Beverly M. Essue, Angela Beaton, Catherine Hull, John Belfrage, Shannon Thompson, Michele Meachen and James A. Gillespie
Year: 2015
Record Number: 9

|  | Yes | No | Unclear | Not applicable |
| --- | --- | --- | --- | --- |
| 1. Is there congruity between the stated philosophical perspective and the research methodology?   Yes, there is congruity; the study employs a pragmatic, mixed-methods approach to capture both the quantitative and qualitative dimensions of economic hardship, which aligns with a perspective that values comprehensive, real-world understanding of complex issues. | □ | □ | □ | □ |
| 1. Is there congruity between the research methodology and the research question or objectives?   Yes, the mixed-methods, prospective case study design is well-suited to the dual objectives of measuring out-of-pocket costs and exploring factors contributing to economic hardship among patients with cancer at the end of life. | □ | □ | □ | □ |
| 1. Is there congruity between the research methodology and the methods used to collect data?   Yes, the combination of structured and semi-structured interviews, along with prospective cost diaries, fits the mixed-methods design and supports both quantitative measurement and qualitative exploration. | □ | □ | □ | □ |
| 1. Is there congruity between the research methodology and the representation and analysis of data?   Yes, quantitative data were analyzed statistically, and qualitative data were thematically analysed, both of which are appropriate for the mixed-methods approach and allow for integrated insights. | □ | □ | □ | □ |
| 1. Is there congruity between the research methodology and the interpretation of results?   Yes, the results are interpreted through both statistical findings and thematic insights, reflecting the mixed-methods design and providing a multidimensional understanding of economic hardship. | □ | □ | □ | □ |
| 1. Is there a statement locating the researcher culturally or theoretically?   No, the study does not explicitly locate the researchers culturally or theoretically, though the interdisciplinary team and their affiliations are described. | □ | □ | □ | □ |
| 1. Is the influence of the researcher on the research, and vice- versa, addressed?   No, there is no explicit discussion of reflexivity or the potential influence of the researchers on the research process or findings. | □ | □ | □ | □ |
| 1. Are participants, and their voices, adequately represented?   Yes, participants’ perspectives are included through direct quotations and thematic analysis, ensuring their experiences and voices are present in the results. | □ | □ | □ | □ |
| 1. Is the research ethical according to current criteria or, for recent studies, and is there evidence of ethical approval by an appropriate body?   Yes, the study received ethics approval from relevant committees and obtained written informed consent from all participants. | □ | □ | □ | □ |
| 1. Do the conclusions drawn in the research report flow from the analysis, or interpretation, of the data?   Yes, the conclusions about the multidimensional nature of economic hardship and the need for nuanced policy responses are well-supported by both the quantitative and qualitative analyses. | □ | □ | □ | □ |

Overall appraisal: Include □ Exclude □ Seek further info □

**Comments (Including reason for exclusion):**

In principle, the study meets the essential requirements. However, two aspects (points 6 and 7) remain unclear: the researchers did not provide any statements regarding their beliefs and values or how these may have influenced the study. Furthermore, it is unclear how the relationship between the researchers and the study participants was addressed.

JBI Critical Appraisal Checklist for
Qualitative Research

Reviewer: Gerald Michelak Date: 17.01.2025

Author: Melissa Giesbrecht, Faye Wolse, Valorie A. Crooks and Kelli Stajduhar
Year: 2015
Record Number: 10

|  | Yes | No | Unclear | Not applicable |
| --- | --- | --- | --- | --- |
| 1. Is there congruity between the stated philosophical perspective and the research methodology?   Yes, there is congruity; the study adopts an intersectionality lens and a qualitative case study approach, both of which align with a constructivist philosophical perspective that values complexity and context in understanding resilience. | □ | □ | □ | □ |
| 1. Is there congruity between the research methodology and the research question or objectives?   Yes, the qualitative case study methodology is well-matched to the objective of exploring socio-environmental factors influencing caregiver resilience, allowing for in-depth examination of complex, context-dependent phenomena. | □ | □ | □ | □ |
| 1. Is there congruity between the research methodology and the methods used to collect data?   Yes, the use of ethnographic fieldnotes and semi-structured interviews fits the qualitative case study methodology, enabling rich, detailed data collection about lived experiences. | □ | □ | □ | □ |
| 1. Is there congruity between the research methodology and the representation and analysis of data?   Yes, the thematic analysis of interview and observational data, guided by an intersectionality lens, is consistent with qualitative case study methodology and supports nuanced interpretation. | □ | □ | □ | □ |
| 1. Is there congruity between the research methodology and the interpretation of results?   Yes, the interpretation of results emphasizes the multidimensional and context-dependent nature of resilience, reflecting the methodological and philosophical underpinnings of the study. | □ | □ | □ | □ |
| 1. Is there a statement locating the researcher culturally or theoretically?   Unclear, although the researchers state their use of an intersectionality lens and critical standpoint perspective, they have not mentioned their theoretical position within the analysis. | □ | □ | □ | □ |
| 1. Is the influence of the researcher on the research, and vice- versa, addressed?   The researchers did not describe how their backgrounds may have influenced the data collection or analysis. | □ | □ | □ | □ |
| 1. Are participants, and their voices, adequately represented?   Yes, participants’ perspectives are presented through direct quotes and detailed case descriptions, ensuring their voices are central to the findings. | □ | □ | □ | □ |
| 1. Is the research ethical according to current criteria or, for recent studies, and is there evidence of ethical approval by an appropriate body?   Yes, the study reports obtaining ethics approval from university and health authority boards and describes informed consent procedures. | □ | □ | □ | □ |
| 1. Do the conclusions drawn in the research report flow from the analysis, or interpretation, of the data?   Yes, the conclusions about the role of socio-environmental factors in caregiver resilience are directly supported by the thematic analysis of the qualitative data. | □ | □ | □ | □ |

Overall appraisal: Include □ Exclude □ Seek further info □

**Comments (Including reason for exclusion):**

In principle, the study meets the essential requirements. However, two aspects (point 6 and 7) remain unclear: it is unclear how the relationship between the researchers and the study participants was addressed. It was merely stated that nurses were responsible for recruiting family caregivers and care recipients whose health status they considered sufficiently stable. The researchers were guided in recording their field notes through the use of an observation guide.

JBI Critical Appraisal Checklist for
Qualitative Research

Reviewer: Gerald Michelak Date: 17.01.2025

Author: Barbara Hanratty, Elizabeth Lowson, Louise Holmes, Gunn Grand, Julia Addington-Hall, Sheila Payne and Jane Seymour
Year: 2012
Record Number: 11

|  | Yes | No | Unclear | Not applicable |
| --- | --- | --- | --- | --- |
| 1. Is there congruity between the stated philosophical perspective and the research methodology?   Yes, there is congruity; the study is grounded in an interpretivist perspective, aiming to understand the subjective views of older adults, and employs qualitative interviews, which are appropriate for exploring individual experiences and perspectives. | □ | □ | □ | □ |
| 1. Is there congruity between the research methodology and the research question or objectives?   Yes, the qualitative interview methodology is well-suited to the study’s objective of exploring older adults’ views on funding health and social care and their related expenditures. | □ | □ | □ | □ |
| 1. Is there congruity between the research methodology and the methods used to collect data?   Yes, in-depth semi-structured interviews are consistent with qualitative research and allow for detailed exploration of participants’ beliefs and experiences. | □ | □ | □ | □ |
| 1. Is there congruity between the research methodology and the representation and analysis of data?   Yes, the use of the matrix-based Framework approach for thematic analysis aligns with qualitative methodology and supports systematic identification and interpretation of key themes. | □ | □ | □ | □ |
| 1. Is there congruity between the research methodology and the interpretation of results?   Yes, the interpretation of results is grounded in the themes that emerged from the data and reflects the qualitative approach of the methodology. | □ | □ | □ | □ |
| 1. Is there a statement locating the researcher culturally or theoretically?   No, while the researchers do not explicitly state their own cultural or theoretical positioning, the study’s focus and design suggest an awareness of the social context and values of the participants. | □ | □ | □ | □ |
| 1. Is the influence of the researcher on the research, and vice- versa, addressed?   No, the study does not explicitly discuss reflexivity or how the researchers’ perspectives may have influenced data collection or analysis. | □ | □ | □ | □ |
| 1. Are participants, and their voices, adequately represented?   Yes, participants’ voices are well-represented through direct quotations and detailed descriptions of their views, ensuring their perspectives are central to the findings. | □ | □ | □ | □ |
| 1. Is the research ethical according to current criteria or, for recent studies, and is there evidence of ethical approval by an appropriate body?   Yes, ethical approval was obtained from the relevant committee, and informed written consent was secured from all participants. | □ | □ | □ | □ |
| 1. Do the conclusions drawn in the research report flow from the analysis, or interpretation, of the data?   Yes, the conclusions are directly supported by the thematic analysis and reflect the participants’ expressed views and experiences. | □ | □ | □ | □ |

Overall appraisal: Include □ Exclude □ Seek further info □

**Comments (Including reason for exclusion):**

In principle, the study meets the essential requirements. However, two aspects (points 6 and 7) remain unclear: the researchers did not provide any statements regarding their beliefs and values or how these may have influenced the study. Furthermore, it is unclear how the relationship between the researchers and the study participants was addressed.

JBI Critical Appraisal Checklist for
Qualitative Research

Reviewer: Gerald Michelak Date: 17.01.2025

Author: Franziska A. Herbst, Laura Gawinski, Nils Schneider and Stephanie Stiel
Year: 2013
Record Number: 12

|  | Yes | No | Unclear | Not applicable |
| --- | --- | --- | --- | --- |
| 1. Is there congruity between the stated philosophical perspective and the research methodology?   The study uses a convergent parallel mixed-methods design with an exploratory, constructivist perspective, which aligns well with the aim to understand subjective support experiences in dyads. Both qualitative and quantitative approaches are justified and complement each other. | □ | □ | □ | □ |
| 1. Is there congruity between the research methodology and the research question or objectives?   The convergent parallel mixed-methods design fits the research objective of capturing detailed support experiences and needs in adult child–parent dyads at the end of life. The methodology enables a comprehensive view of the phenomenon. | □ | □ | □ | □ |
| 1. Is there congruity between the research methodology and the methods used to collect data?   Semi-structured interviews and validated questionnaires (Berlin Social Support Scales) are appropriate for the mixed-methods approach. These methods allow for both in-depth exploration and quantification of support experiences. | □ | □ | □ | □ |
| 1. Is there congruity between the research methodology and the representation and analysis of data?   Qualitative data were analyzed using a grounded theory approach, and quantitative data were statistically evaluated, with results integrated in the discussion. This approach matches the mixed-methods design and ensures robust representation of findings. | □ | □ | □ | □ |
| 1. Is there congruity between the research methodology and the interpretation of results?   Interpretation of results is grounded in both qualitative themes and quantitative findings, with hypotheses and recommendations clearly linked to the data. The conclusions are supported by the integrated analysis. | □ | □ | □ | □ |
| 1. Is there a statement locating the researcher culturally or theoretically?   Author’s biographies are described, but there is no explicit reflection on their cultural or theoretical positions. Personal beliefs or values are not discussed. | □ | □ | □ | □ |
| 1. Is the influence of the researcher on the research, and vice- versa, addressed?   The study mentions independent coding and consensus-building within the team but does not explicitly discuss how researchers’ perspectives may have influenced the process. Reflexivity is not deeply addressed. | □ | □ | □ | □ |
| 1. Are participants, and their voices, adequately represented?   Participants’ voices are well represented through direct quotes and detailed qualitative findings. The diversity of experiences within both dyad types is clearly illustrated. | □ | □ | □ | □ |
| 1. Is the research ethical according to current criteria or, for recent studies, and is there evidence of ethical approval by an appropriate body?   Ethical approval was obtained, informed consent was ensured, and participant well-being was prioritized throughout the study. Confidentiality and voluntary participation are documented. | □ | □ | □ | □ |
| 1. Do the conclusions drawn in the research report flow from the analysis, or interpretation, of the data?   Conclusions and recommendations are logically derived from the integrated qualitative and quantitative data analysis. The findings are well substantiated by the presented results. | □ | □ | □ | □ |

Overall appraisal: Include □ Exclude □ Seek further info □

**Comments (Including reason for exclusion):**

In principle, the study meets the essential requirements. However, two aspects (points 6 and 7) remain unclear: the researchers did not provide any statements regarding their beliefs and values or how these may have influenced the study. Furthermore, it is unclear how the relationship between the researchers and the study participants was addressed.

JBI Critical Appraisal Checklist for
Qualitative Research

Reviewer: Gerald Michelak Date: 21.01.2025

Author: Sarina R. Isenberg, Tieghan Killackey, Stephanie Saunders, Mary Scott, Natalie C. Ernecoff, Shirley H. Bush, Jaymie Varenbut, Emily Lovrics, Maya A. Stern, Amy T. Hsu, Mark Bernstein and Camilla Zimmermann
Year: 2021
Record Number: 13

|  | Yes | No | Unclear | Not applicable |
| --- | --- | --- | --- | --- |
| 1. Is there congruity between the stated philosophical perspective and the research methodology?   The study employs a constructivist, grounded theory approach to explore subjective experiences of hospital-to-home transitions, which aligns well with the aim of developing a substantive theory in this context. | □ | □ | □ | □ |
| 1. Is there congruity between the research methodology and the research question or objectives?   The longitudinal qualitative design is appropriate for exploring both expectations and actual experiences of patients and caregivers over time, as well as for theory development. | □ | □ | □ | □ |
| 1. Is there congruity between the research methodology and the methods used to collect data?   Semi-structured interviews at two time points (before discharge and several weeks after) are well suited to grounded theory and allow for capturing evolving perspectives. | □ | □ | □ | □ |
| 1. Is there congruity between the research methodology and the representation and analysis of data?   Data were analyzed using open and axial coding in line with grounded theory, with iterative development of a codebook and consensus coding, ensuring systematic and robust analysis. | □ | □ | □ | □ |
| 1. Is there congruity between the research methodology and the interpretation of results?   Interpretation of results is closely linked to the emergent themes and participant narratives, and the developed theory is grounded in the data. | □ | □ | □ | □ |
| 1. Is there a statement locating the researcher culturally or theoretically?   The researchers’ institutional affiliations and professional backgrounds are described, but there is no explicit reflection on their cultural or theoretical positions or personal values. | □ | □ | □ | □ |
| 1. Is the influence of the researcher on the research, and vice- versa, addressed?   The study notes that interviewers had no prior relationship with participants and describes consensus coding and memo writing but does not deeply discuss researcher influence or reflexivity. | □ | □ | □ | □ |
| 1. Are participants, and their voices, adequately represented?   Participants’ voices are well represented through direct quotations and detailed thematic illustration, providing insight into both patient and caregiver perspectives. | □ | □ | □ | □ |
| 1. Is the research ethical according to current criteria or, for recent studies, and is there evidence of ethical approval by an appropriate body?   Ethical approval was obtained from relevant boards, informed consent was ensured, and participant well-being and confidentiality were prioritized. | □ | □ | □ | □ |
| 1. Do the conclusions drawn in the research report flow from the analysis, or interpretation, of the data?   Conclusions and recommendations are logically derived from the data analysis and are supported by the developed substantive grounded theory and participant experiences. | □ | □ | □ | □ |

Overall appraisal: Include □ Exclude □ Seek further info □

**Comments (Including reason for exclusion):**

In principle, the study meets the essential requirements. However, two aspects (points 6 and 7) remain unclear: the researchers did not provide any statements regarding their beliefs and values or how these may have influenced the study. Furthermore, it is unclear how the relationship between the researchers and the study participants was addressed.

JBI Critical Appraisal Checklist for
Qualitative Research

Reviewer: Daniela Bernhardsgrütter Date: 10.11.2025

Author: Christiane Kreyer, Barbara Stecher, Sabine Pleschberger and Gail Ewing
Year: 2024
Record Number: 14

|  | Yes | No | Unclear | Not applicable |
| --- | --- | --- | --- | --- |
| 1. Is there congruity between the stated philosophical perspective and the research methodology?   The philosophical perspective is not mentioned. | □ | □ | □ | □ |
| 1. Is there congruity between the research methodology and the research question or objectives?   The retrospective study design using qualitative and quantitative methods aligns with research aims. | □ | □ | □ | □ |
| 1. Is there congruity between the research methodology and the methods used to collect data?   The retrospective analysis of electronic records aligns with the research methodology. | □ | □ | □ | □ |
| 1. Is there congruity between the research methodology and the representation and analysis of data?   Content analysis (deductive and inductive coding) aligns with research methodology. | □ | □ | □ | □ |
| 1. Is there congruity between the research methodology and the interpretation of results?   Interpretations are derived from the categorized and analyzed data, matching the study approach | □ | □ | □ | □ |
| 1. Is there a statement locating the researcher culturally or theoretically?   The authors’ theoretical background is stated. | □ | □ | □ | □ |
| 1. Is the influence of the researcher on the research, and vice- versa, addressed?   Two authors conducted data analysis and discussed results within the research team. | □ | □ | □ | □ |
| 1. Are participants, and their voices, adequately represented?   Themes are illustrated in detail. No quotes needed because no interviews were conducted. | □ | □ | □ | □ |
| 1. Is the research ethical according to current criteria or, for recent studies, and is there evidence of ethical approval by an appropriate body?  \| Ethical approval was obtained. \| \| --- \| | □ | □ | □ | □ |
| 1. Do the conclusions drawn in the research report flow from the analysis, or interpretation, of the data?   Conclusions are based on findings and interpretation. | □ | □ | □ | □ |

Overall appraisal: Include □ Exclude □ Seek further info □

**Comments (Including reason for exclusion):**

The study meets the essential requirements. The philosophical perspective is not mentioned.

JBI Critical Appraisal Checklist for
Qualitative Research - DISSERTATION

Reviewer: Daniela Bernhardsgrütter Date: 06.11.2025

Author: Joanne Lewis
Year: 2011
Record Number: 15

|  | Yes | No | Unclear | Not applicable |
| --- | --- | --- | --- | --- |
| 1. Is there congruity between the stated philosophical perspective and the research methodology?   The study adopts a pragmatic paradigm, which aligns with the mixed methods approach. | □ | □ | □ | □ |
| 1. Is there congruity between the research methodology and the research question or objectives?   The mixed methods design is suitable for exploring the complexity of the research topic. It aligns with the research aims, which address both qualitative aspects (experiences) and quantitative aspects (e.g., the impact of socioeconomic status on place of death and service utilization). | □ | □ | □ | □ |
| 1. Is there congruity between the research methodology and the methods used to collect data?   The use of semi-structured in-depth interview, self-report tools and administrative data aligns with the mixed methods design. | □ | □ | □ | □ |
| 1. Is there congruity between the research methodology and the representation and analysis of data?   The framework approach to qualitative data analysis is suitable for mixed methods studies, providing a structured approach to qualitatively analyse complex data. | □ | □ | □ | □ |
| 1. Is there congruity between the research methodology and the interpretation of results?   The author provided rich descriptions of themes, supported by participants’ quotations. | □ | □ | □ | □ |
| 1. Is there a statement locating the researcher culturally or theoretically?   The cultural and theoretical background of the author was stated. | □ | □ | □ | □ |
| 1. Is the influence of the researcher on the research, and vice- versa, addressed?   Positioning of the researcher is addressed thoroughly. | □ | □ | □ | □ |
| 1. Are participants, and their voices, adequately represented?   Numerous direct quotations illustrate themes clearly and authentically. | □ | □ | □ | □ |
| 1. Is the research ethical according to current criteria or, for recent studies, and is there evidence of ethical approval by an appropriate body?  \| Ethical approval, informed consent, and protection of vulnerable participants described. \| \| --- \| | □ | □ | □ | □ |
| 1. Do the conclusions drawn in the research report flow from the analysis, or interpretation, of the data?   Themes well-grounded in verbatim quotes; coherence between data and conclusions. | □ | □ | □ | □ |

Overall appraisal: Include □ Exclude □ Seek further info □

**Comments (Including reason for exclusion):**

The study fully meets the essential requirements, all aspects were elaborated in detail.

JBI Critical Appraisal Checklist for
Qualitative Research

Reviewer: Daniela Bernhardsgrütter Date: 10.11.2025

Author: Geck Hoon Lim, Celine Yong, Lauren J. Breen, Sharon Keesing and Angus Buchanan
Year: 2024
Record Number: 16

|  | Yes | No | Unclear | Not applicable |
| --- | --- | --- | --- | --- |
| 1. Is there congruity between the stated philosophical perspective and the research methodology?   The adopted qualitative exploratory approach aligns with the constructivist paradigm. | □ | □ | □ | □ |
| 1. Is there congruity between the research methodology and the research question or objectives?   Exploring experiences with occupations aligns with the qualitative exploratory approach. | □ | □ | □ | □ |
| 1. Is there congruity between the research methodology and the methods used to collect data?   Data collection methods are well described and appropriate for qualitative inquiry. | □ | □ | □ | □ |
| 1. Is there congruity between the research methodology and the representation and analysis of data?   Reflexive thematic analysis aligns with qualitative exploratory approach | □ | □ | □ | □ |
| 1. Is there congruity between the research methodology and the interpretation of results?   The author provided rich descriptions of themes, supported by participants’ quotations. | □ | □ | □ | □ |
| 1. Is there a statement locating the researcher culturally or theoretically?   The theoretical background of the authors was stated. | □ | □ | □ | □ |
| 1. Is the influence of the researcher on the research, and vice- versa, addressed?   Positioning of the researcher is addressed thoroughly. | □ | □ | □ | □ |
| 1. Are participants, and their voices, adequately represented?   Numerous direct quotations illustrate themes clearly and authentically. | □ | □ | □ | □ |
| 1. Is the research ethical according to current criteria or, for recent studies, and is there evidence of ethical approval by an appropriate body?  \| Ethical approval was obtained. \| \| --- \| | □ | □ | □ | □ |
| 1. Do the conclusions drawn in the research report flow from the analysis, or interpretation, of the data?   Themes well-grounded in verbatim quotes; coherence between data and conclusions. | □ | □ | □ | □ |

Overall appraisal: Include □ Exclude □ Seek further info □

**Comments (Including reason for exclusion):**

The study meets the essential requirements, but descriptions of data collections methods and tools (e.g. interview guide) are not precise.

JBI Critical Appraisal Checklist for
Qualitative Research

Reviewer: Marlene Matzinger Date: 02.11.2025

Author: Sally Pieper, Alina Kasdorf, Raymond Voltz and Julia Strupp
Year: 2025
Record Number: 17

|  | Yes | No | Unclear | Not applicable |
| --- | --- | --- | --- | --- |
| 1. Is there congruity between the stated philosophical perspective and the research methodology?   Unclear. The overarching philosophical perspective was not stated. | □ | □ | □ | □ |
| 1. Is there congruity between the research methodology and the research question or objectives?   Yes. The research question regarding the financial impact of caring for a seriously ill and dying family member is clearly described and aligns with the applied qualitative methodology. | □ | □ | □ | □ |
| 1. Is there congruity between the research methodology and the methods used to collect data?   Yes. Data were collected using an online survey with open-ended text fields as well as a validated needs assessment scale (CSNAT/KOMMA), which were systematically analyzed qualitatively. | □ | □ | □ | □ |
| 1. Is there congruity between the research methodology and the representation and analysis of data?   Yes. The data were analyzed using content analysis. The open-text responses were coded and then analyzed. A category system with main and subcategories was developed. | □ | □ | □ | □ |
| 1. Is there congruity between the research methodology and the interpretation of results?   Yes, the interpretation reflects the data and refers to the participants’ categories and quotes. | □ | □ | □ | □ |
| 1. Is there a statement locating the researcher culturally or theoretically?   No. There is no explicit reflection on their cultural or theoretical positions or on how these may have influenced the study. | □ | □ | □ | □ |
| 1. Is the influence of the researcher on the research, and vice- versa, addressed?   The study describes team collaboration and consensus-building but does not provide a detailed reflection on potential researcher assumptions or the influence of the researchers on the research process. | □ | □ | □ | □ |
| 1. Are participants, and their voices, adequately represented?   Participants’ voices are adequately represented in the results through numerous quotes | □ | □ | □ | □ |
| 1. Is the research ethical according to current criteria or, for recent studies, and is there evidence of ethical approval by an appropriate body?   Yes, the study was approved by the Ethics Committee of the Medical Faculty of Cologne, participant consent is reported. | □ | □ | □ | □ |
| 1. Do the conclusions drawn in the research report flow from the analysis, or interpretation, of the data?  Yes, the conclusions are linked to the themes and findings that emerged from the data analysis. | □ | □ | □ | □ |

Overall appraisal: Include □ Exclude □ Seek further info □

Comments (Including reason for exclusion)

In principle, the study meets the essential requirements. However, the researchers did not provide any statements regarding their beliefs and values or how these may have influenced the study. Furthermore, it is unclear how the relationship between the researchers and the study participants was addressed and the overarching philosophical perspective not mentioned.

JBI Critical Appraisal Checklist for
Qualitative Research

Reviewer: Marlene Matzinger Date: 03.11.2025

Author: Simona Sacchi, Loredana Buonaccorso, Silvia Tanzi, Giulietta Luul Balestra and Luca Ghirotto
Year: 2025
Record Number: 18

|  | Yes | No | Unclear | Not applicable |
| --- | --- | --- | --- | --- |
| 1. Is there congruity between the stated philosophical perspective and the research methodology?   The grounded theory was consistently applied: theoretical sampling, semi-structured interviews, iterative coding (initial, focused, theoretical), reflexivity, were described and practiced. | □ | □ | □ | □ |
| 1. Is there congruity between the research methodology and the research question or objectives?   The research question (‘What influences decision-making processes regarding the place of death among migrants with cancer?’) is addressed using a constructivist grounded theory approach. The methodology is consistent with the research question. | □ | □ | □ | □ |
| 1. Is there congruity between the research methodology and the methods used to collect data?   The chosen methodology (in-depth semi-structured interviews) is appropriate for grounded theory and aligns with the study’s objectives. | □ | □ | □ | □ |
| 1. Is there congruity between the research methodology and the representation and analysis of data?   The data analysis follows Charmaz: iterative coding by multiple researchers, consensus-building, and transparent reflexivity were explicitly applied. Memos document the influence of researcher characteristics and cultural mediation on interpretation. | □ | □ | □ | □ |
| 1. Is there congruity between the research methodology and the interpretation of results?   The interpretation of results in the study is fully congruent with the constructivist grounded theory methodology, as findings were derived through iterative coding and constant comparison in line with Charmaz’s approach. | □ | □ | □ | □ |
| 1. Is there a statement locating the researcher culturally or theoretically?   Yes, the study explicitly locates the research team both culturally and theoretically. The authors describe their multidisciplinary composition—two palliative care physicians, a psychologist/psychotherapist, a medical anthropologist, and a qualitative methodologist/ sociologist. Importantly, they reflect on how their positionalities (such as gender, linguistic background, and lack of direct migration experience) might have influenced both data collection and interpretation | □ | □ | □ | □ |
| 1. Is the influence of the researcher on the research, and vice- versa, addressed?   The authors describe their own positions (no migration background, various disciplines) and continuously reflect on potential influences on data collection and interpretation. | □ | □ | □ | □ |
| 1. Are participants, and their voices, adequately represented?   Yes, participants and their voices are adequately represented—many direct quotes and case descriptions from patients, family members, and key informants illustrate their lived experiences, concerns, and perspectives throughout the findings section, reflecting diversity and authenticity. | □ | □ | □ | □ |
| 1. Is the research ethical according to current criteria or, for recent studies, and is there evidence of ethical approval by an appropriate body?   The Provincial Ethics Committee of Reggio Emilia approved the initial study. Participants provided oral and written informed consent, including authorization for sensitive data processing. | □ | □ | □ | □ |
| 1. Do the conclusions drawn in the research report flow from the analysis, or interpretation, of the data?   Yes, the conclusions in the research report clearly flow from the analysis and interpretation of the data. The conceptual model and recommendations are derived directly from the grounded theory coding, thematic categories, and participant narratives presented in the results section. | □ | □ | □ | □ |

Overall appraisal: Include □ Exclude □ Seek further info □

Comments (Including reason for exclusion)

The study demonstrates full congruity between philosophical perspective (constructivist grounded theory), methodology, methods, analysis, interpretation, researcher reflexivity, participant representation, ethics, and conclusions. All steps align and are transparently described.

JBI Critical Appraisal Checklist for
Qualitative Research

Reviewer: Gerald Michelak Date: 21.01.2025

Author: Diane N. Solomon and Lissi Hansen
Year: 2013
Record Number: 19

|  | Yes | No | Unclear | Not applicable |
| --- | --- | --- | --- | --- |
| 1. Is there congruity between the stated philosophical perspective and the research methodology?   The study uses Benner’s interpretive phenomenological approach, which is well aligned with the aim to explore the lived experiences and embedded meanings of dying at home. | □ | □ | □ | □ |
| 1. Is there congruity between the research methodology and the research question or objectives?   The phenomenological single-case design fits the objective to deeply understand one family’s experience and the influence of home death on bereavement patterns. | □ | □ | □ | □ |
| 1. Is there congruity between the research methodology and the methods used to collect data?   Separate, in-depth interviews with the patient and family members are appropriate for phenomenological inquiry, allowing for rich, detailed accounts. | □ | □ | □ | □ |
| 1. Is there congruity between the research methodology and the representation and analysis of data?   Data were analyzed using paradigm cases, exemplars, and thematic analysis, consistent with Benner’s phenomenological method and ensuring meaningful pattern identification. | □ | □ | □ | □ |
| 1. Is there congruity between the research methodology and the interpretation of results?   Interpretation is closely tied to the themes and paradigm cases that emerged from the data, with findings grounded in participants’ narratives. | □ | □ | □ | □ |
| 1. Is there a statement locating the researcher culturally or theoretically?   The researchers’ professional backgrounds are described, but there is no explicit reflection on their cultural or theoretical positions or on how these may have influenced the study. | □ | □ | □ | □ |
| 1. Is the influence of the researcher on the research, and vice- versa, addressed?   The principal investigator had a pre-existing relationship with the family, which is mentioned, but the influence of this relationship on data collection and interpretation is not deeply discussed. | □ | □ | □ | □ |
| 1. Are participants, and their voices, adequately represented?   Participants’ voices are well represented through extensive direct quotations and detailed narrative accounts, highlighting both patient and family perspectives. | □ | □ | □ | □ |
| 1. Is the research ethical according to current criteria or, for recent studies, and is there evidence of ethical approval by an appropriate body?   Ethical approval was obtained, informed consent was clearly described, and confidentiality and participant well-being were prioritized. | □ | □ | □ | □ |
| 1. Do the conclusions drawn in the research report flow from the analysis, or interpretation, of the data?   Conclusions about the meaning of dying at home and its impact on bereavement are logically derived from the thematic analysis and are substantiated by the data. | □ | □ | □ | □ |

Overall appraisal: Include □ Exclude □ Seek further info □

**Comments (Including reason for exclusion):**

In principle, the study meets the essential requirements. However, two aspects (points 6 and 7) remain unclear: the researchers did not provide any statements regarding their beliefs and values or how these may have influenced the study. Furthermore, it is unclear how the relationship between the researchers and the study participants was addressed.

JBI Critical Appraisal Checklist for
Qualitative Research

Reviewer: Gerald Michelak Date: 23.01.2025

Author: Deborah P. Waldrop, Betty J. Kramer, Judith A. Skretny, Robert A. Milch and William Finn
Year: 2005
Record Number: 20

|  | Yes | No | Unclear | Not applicable |
| --- | --- | --- | --- | --- |
| 1. Is there congruity between the stated philosophical perspective and the research methodology?   Yes. The study adopts a qualitative, interpretive approach consistent with its aim to understand caregivers' experiences using the stress process model. | □ | □ | □ | □ |
| 1. Is there congruity between the research methodology and the research question or objectives?   Yes. The use of in-depth qualitative interviews aligns well with the objective to explore the unique aspects of end-stage caregiving transitions. | □ | □ | □ | □ |
| 1. Is there congruity between the research methodology and the methods used to collect data?   Yes. Qualitative in-depth interviews are appropriate for a methodology focused on exploring personal experiences and emergent themes. | □ | □ | □ | □ |
| 1. Is there congruity between the research methodology and the representation and analysis of data?   Yes. Data were analyzed using constant comparative analysis, which is suitable for qualitative research and supports theme development. | □ | □ | □ | □ |
| 1. Is there congruity between the research methodology and the interpretation of results?   Yes. The interpretation of results is grounded in the emergent themes and directly reflects the qualitative data collected from caregivers. | □ | □ | □ | □ |
| 1. Is there a statement locating the researcher culturally or theoretically?   No, the researcher did not describe their cultural or theoretical background. | □ | □ | □ | □ |
| 1. Is the influence of the researcher on the research, and vice- versa, addressed?   No. The study does not discuss reflexivity or the potential influence of the researchers on the data or analysis. | □ | □ | □ | □ |
| 1. Are participants, and their voices, adequately represented?   Yes. The study presents caregivers' experiences and quotations, ensuring that participants' voices are central to the findings. | □ | □ | □ | □ |
| 1. Is the research ethical according to current criteria or, for recent studies, and is there evidence of ethical approval by an appropriate body?   The study describes a careful recruitment protocol to avoid distress but there is explicit mention of ethical approval. | □ | □ | □ | □ |
| 1. Do the conclusions drawn in the research report flow from the analysis, or interpretation, of the data?   Yes. The conclusions are clearly linked to the themes and findings that emerged from the data analysis. | □ | □ | □ | □ |

Overall appraisal: Include □ Exclude □ Seek further info □

**Comments (Including reason for exclusion):**

In principle, the study meets the essential requirements. However, three aspects (points 6, 7, 9) remain unclear: the researchers did not provide any statements regarding their beliefs and values or how these may have influenced the study. Furthermore, it is unclear how the relationship between the researchers and the study participants was addressed and if there was ethical approval by an appropriate body.
